# Visual speech discrimination and identification of natural and synthetic consonant stimuli

**DOI:** 10.3389/fpsyg.2015.00878

**Published:** 2015-07-13

**Authors:** Benjamin T. Files, Bosco S. Tjan, Jintao Jiang, Lynne E. Bernstein

**Affiliations:** ^1^U.S. Army Research Laboratory, Human Research and Engineering Directorate, Aberdeen Proving GroundMD, USA; ^2^Department of Psychology, University of Southern California, Los AngelesCA, USA; ^3^Applications Technology, McLeanVA, USA; ^4^Department of Speech and Hearing Science, George Washington University, WashingtonDC, USA

**Keywords:** visual speech perception, visemes, lipreading/speechreading, discrimination, synthetic visual speech, motion capture, multisensory perception, audiovisual speech perception

## Abstract

From phonetic features to connected discourse, every level of psycholinguistic structure including prosody can be perceived through viewing the talking face. Yet a longstanding notion in the literature is that visual speech perceptual categories comprise groups of phonemes (referred to as *visemes*), such as /p, b, m/ and /f, v/, whose internal structure is not informative to the visual speech perceiver. This conclusion has not to our knowledge been evaluated using a psychophysical discrimination paradigm. We hypothesized that perceivers can discriminate the phonemes within typical viseme groups, and that discrimination measured with *d-prime* (*d’*) and response latency is related to visual stimulus dissimilarities between consonant segments. In Experiment 1, participants performed speeded discrimination for pairs of consonant-vowel spoken nonsense syllables that were predicted to be *same, near*, or *far* in their perceptual distances, and that were presented as natural or synthesized video. *Near* pairs were within-viseme consonants. Natural within-viseme stimulus pairs were discriminated significantly above chance (except for /k/-/h/). *S*ensitivity (*d’*) increased and response times decreased with distance. Discrimination and identification were superior with natural stimuli, which comprised more phonetic information. We suggest that the notion of the viseme as a unitary perceptual category is incorrect. Experiment 2 probed the perceptual basis for visual speech discrimination by inverting the stimuli. Overall reductions in *d’* with inverted stimuli but a persistent pattern of larger *d’* for *far* than for *near* stimulus pairs are interpreted as evidence that visual speech is represented by both its motion and configural attributes. The methods and results of this investigation open up avenues for understanding the neural and perceptual bases for visual and audiovisual speech perception and for development of practical applications such as visual lipreading/speechreading speech synthesis.

## Introduction

Visual speech perception (i.e., also known as *lipreading* or *speechreading*) relies exclusively on visible information from a talking face. Published literature suggests that every level of psycholinguistic speech structure (e.g., phonetic features, phonemes, syllables, words, and prosody) is visible (Bernstein and Liebenthal, 2014), although there are individual differences in the ability to perceive the information in visual speech (Bernstein et al., 2000; Auer and Bernstein, 2007). For example, deaf lipreaders scored between zero and 85% correct on a test of lipreading words in sentences, and hearing lipreaders scored between zero and 75% correct (Bernstein et al., 2000). Isolated monosyllabic word identification with highly similar rhyming words was as high as 42% correct in deaf lipreaders and 24% correct in hearing lipreaders (see also, Conklin, 1917; Utley, 1946; Lyxell et al., 1993; Auer, 2002; Mattys et al., 2002). There is evidence that phonemes and phonetic features are visible, again with perceptual accuracy varying across factors such as the talker, the stimulus materials, the perceiver, and the perceiver group (e.g., deaf, hearing, children; Woodward and Barber, 1960; Fisher, 1968; Massaro and Cohen, 1983; Owens and Blazek, 1985; Demorest and Bernstein, 1992; Bernstein et al., 2000, 2001; Jiang et al., 2007; Tye-Murray et al., 2014). Lexical and sentential prosodic distinctions vary in their visibility (Fisher, 1969; Eberhardt et al., 1990; Lansing and McConkie, 1999; Munhall et al., 2004; Scarborough et al., 2007). The study reported here is concerned with the resolution of visual speech phoneme perception, which as we discuss below is widely regarded as poor.

### Visual Speech Phoneme Perception

The visual information in speech derives from the same articulatory organs that drive audible speech, the lips, teeth, tongue, jaw, velum, larynx, and lungs (Catford, 1977); but only the lips, teeth, jaw, and intermittently the tongue are directly visible. Additional face parts such as cheeks and eyebrows can convey speech information that is correlated with the actions of the articulatory organs (Yehia et al., 1998; Lansing and McConkie, 1999; Jiang et al., 2007; Lucero and Munhall, 2008). The invisibility of the larynx and the velum should not be taken as evidence that phonetic features such as voicing are also invisible as phonemic categories are signaled by multiple phonetic cues. For example, vowel duration, a visible feature, is a primary cue to English post-vocalic consonant voicing (Raphael, 1971), and patterns of pre- and post-vocalic consonant perception vary. There is evidence for voicing being visible in syllable-final but not in syllable-initial position (Hnath-Chisolm and Kishon-Rabin, 1988).

Where did the current conception of visual phoneme perception arise? In an early and influential paper on visual speech phoneme perception, Woodward and Barber (1960) reported that there are only four visually contrastive consonantal units available to lipreaders (i.e., /b, p, m/, /hw, w, r/, /f, v/, and all other consonants in the remaining group). Their conclusion was based on a psychophysical method that may not be sufficiently sensitive^1^. Given their small number of derived contrastive units, in order to explain proficient lipreading in deaf individuals, Woodward and Barber (1960) appealed to higher-level language and context. Their results led to research by Fisher (1968), who coined the term *viseme* to describe the units of visual speech perception that he derived from a forced choice word identification task, in which the available responses to be used by participants *did not* include the correct word. Fisher (1968) reported that initial consonant visemes comprise /p, b, m, d/, /f, v/, /k, g/, /hw, w, r/, and, /ʃ, t, n, l, s, z, h, dƷ, tʃ /. Subsequently, Walden et al. (1977) introduced hierarchical clustering analysis to derive viseme groups from data in stimulus-response confusion matrices obtained in a forced-choice identification paradigm. When the clustering threshold was set so that 71% of responses to each phoneme were within a cluster, they obtained nine viseme units (see also, Owens and Blazek, 1985). Subsequently, Auer and Bernstein (1997) generalized the clustering methods to produce a range of within-cluster response thresholds. They introduced the terminology *phoneme equivalence class* (PEC) to designate a family of within-cluster thresholds. The PECs were intended to characterize levels of within-cluster groupings that can be tested against various performance measures, and the PEC was thus not a statement about limitations on discrimination. These authors used PECs to test models of visual spoken word recognition over a range of theoretically derived perceptual category resolutions.

The Auer–Bernstein PEC is a view that runs counter to the traditional and now pervasive notion of the viseme as a perceptual unit (Mattheyses and Verhelst, 2014). Massaro characterized the traditional viseme view saying, “Because of the data-limited property of visible speech in comparison to audible speech, many phonemes are virtually indistinguishable by sight, even from a natural face, and so are expected to be easily confused” (Massaro et al., 2012, p. 316); and that, “a difference between visemes is significant, informative, and categorical to the perceiver; a difference within a viseme class is not” (Massaro et al., 2012, p. 316).

However, there is abundant evidence that viewers are very sensitive to visual speech information. For example, even point-light speech stimuli (only the movement of a few points on the face) can enhance the intelligibility of acoustic speech in noise (Rosenblum et al., 1996) and can interfere with audiovisual speech perception when they are incongruent (Rosenblum and Saldana, 1996). Importantly, visual phonetic information enhances speech perception in acoustically noisy and distorting environments or with hearing loss (Sumby and Pollack, 1954; MacLeod and Summerfield, 1987; Ross et al., 2007; Grant et al., 2013), and subtle differences in individual speech tokens control the type of audiovisual perceptual (Jiang and Bernstein, 2011) and neural interactions (Bernstein et al., 2011) that are obtained with mismatched audiovisual stimuli. In addition, visual speech synthesis is improved when phonetic context-sensitive models are used rather than simpler models that use the same viseme regardless of context (Mattheyses et al., 2013), implying perception of phonetic structure within putative visemes. Indeed, phoneme confusions vary with vowel context, leading to different visemes (Owens and Blazek, 1985).

Recently, Bernstein (2012) introduced direct evidence that sub-viseme phonetic cues are informative. When presented with pairs of spoken words that differed phoneme-by-phoneme within viseme groups, participants (both deaf and normal-hearing adults) were able to identify which of the spoken words corresponded to an orthographic target word (Bernstein, 2012). Target identification remained highly accurate even when word pairs were constructed from PECs comprising hierarchically earlier (more confusable) phonemes than within typical viseme groups. A group of normal-hearing lipreaders scored between 65 and 80% correct, and deaf participants scored between 80 and 100% correct.

To our knowledge, there has been minimal testing of within-viseme perception using a psychophysical discrimination approach, even though the study of auditory phoneme categorization has long been known to require both discrimination and identification paradigms (Liberman et al., 1957). The categorical perception approach was applied by synthesizing continua of visual speech (Walden et al., 1987). But the continua endpoints were highly discriminable between-viseme phonemes, /bɑ/ to /vɑ/ to /wɑ/. Six linearly interpolated (using vector graphics with 130 vectors) stimuli were generated between the consonant-vowel (CV) pairs. Discrimination was significantly more sensitive than that predicted by identification functions. But stimuli were highly artificial, generated using only on an initial closed-mouth video frame, a frame with a maximally articulated consonant, and a single vowel gesture frame with total stimulus duration fixed; and not all of the stimuli appeared to be natural to the participants. In a task requiring discrimination, Massaro et al. (1993) presented CVC words in two-word trials for initial consonant identification with between-viseme phonemes. Performance was near ceiling. A preliminary experiment reported in (Jerger et al., 2009) showed that adults could categorize /p, b, t, d/ as voiced or not voiced at above chance levels, although voicing contrasts with homorganic stops (e.g., /b/ vs. /p/ or /d/ vs. /t/) are considered to be within viseme groups. A recent study showed that adults can also discriminate /b/-/m/ at above chance levels (Lalonde and Holt, 2014).

Recently, we reported on a small set of discrimination results in an electroencephalography (EEG) study (Files et al., 2013) that was designed using a visual mismatch negativity (MMN) paradigm (Winkler and Czigler, 2012) with the intent to obtain a change detection response. Both within- and between-viseme pairs elicited the MMN response.

### The Current Study

**FIGURE 1:**
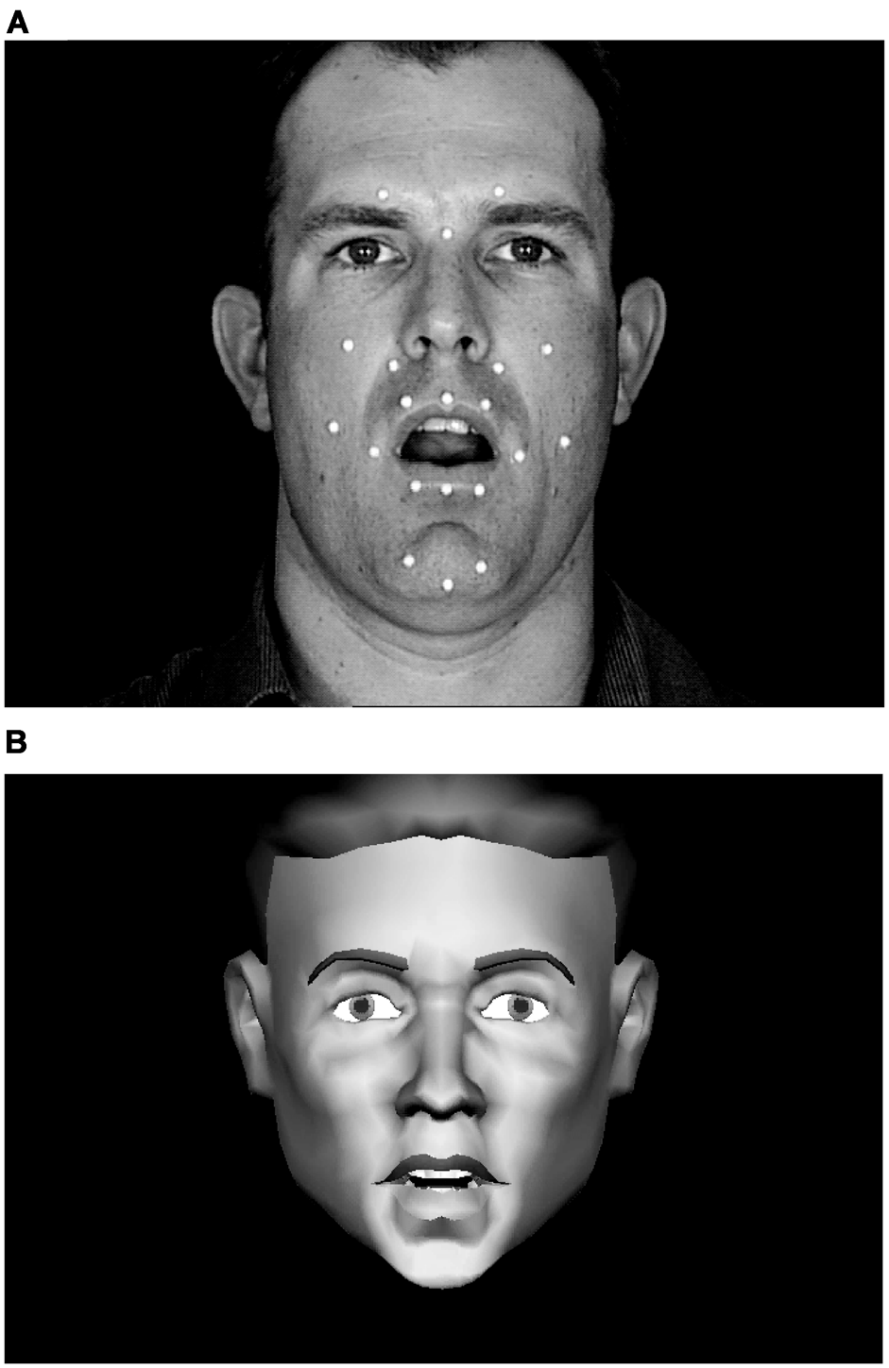
**Still frames from natural and synthetic speech stimuli.** The white dots on the face of the talker **(A)** are retro-reflectors that were used during video recording for motion-capture of 3D motion on the talker’s face. This 3D motion drove the motions of the synthetic talking face **(B)**. Video and synthetic stimuli were presented in full color against a dark blue background.

We carried out a study of visual speech discrimination and identification. Stimulus materials from a single talker were generated in sets of triplets of CV syllables. Out of these sets, stimulus pairs were presented for *same-different* discrimination, and all *same* trials used different tokens of the same phoneme. Seven consonants were designated as anchors for the triplet sets, and each anchor was paired with a perceptually *same, near*, or *far* consonant. The perceptual distance factor was obtained from a previous modeling study (Jiang et al., 2007). Here, *near* stimulus pairs were from within viseme-level PECs, and *far* stimulus pairs were from across visemes-level PECs. In the modeling study (Jiang et al., 2007), CV stimuli (with 23 different initial consonants) were recorded simultaneously with a video camera and a three-dimensional optical recording system. Optical recording tracked the positions of retro-reflectors pasted on the talker’s face (see **Figure 1A**). The video CV stimuli were perceptually identified, and the obtained confusion data were submitted for multidimensional scaling (Kruskal and Wish, 1978) to compute Euclidean distances between stimuli. The three-dimensional motion tracks were also used to calculate optical Euclidean distances. The perceptual distances were used to linearly warp the physical distances using least squares minimization (Kailath et al., 2000). This linear mapping approach was highly successful in accounting for a separate sample of perceptual identification results. The variance in the perceptual distances accounted for by the physical distances ranged between 46 and 66% across the four talkers who were studied and between 49 and 64% across the three vowels (/ɑ/, /i/, and /u/) in the CV stimuli. The syllables in the current study have thus been previously characterized not only in terms of their PEC status but also their perceptual and physical dissimilarities.

In addition, stimuli in Experiment 1 were presented as natural video or synthesized video. Every naturally produced CV utterance is expected to be physically different to some extent, not only in its unique instantiation of a particular CV sequence, but also in terms of other stimulus information such as the talker’s eye gaze, head motion, and possibly subtle differences in talker affect or emotion. Therefore, the current study used two tokens of every phoneme type and also used visual speech synthesis to control for natural variation and to probe the basis for visual speech discrimination. The three-dimensional motion data in the modeling study (Jiang et al., 2007) were used to drive a visual speech wire-frame synthesizer (Jiang et al., 2008). The synthesized speech removed many of the natural non-speech stimulus characteristics that could inflate discrimination. The synthesized talking face image had fixed head and eye positions and no facial emotion or affective changes. We also probed visual speech stimulus features in Experiment 2 by inverting the video stimuli. Inversion disrupts face processing to some extent (Valentine, 1988; Maurer et al., 2002; Richler et al., 2011; Gold et al., 2012).

## Materials and Methods

### Experiment 1: Discrimination and Identification of Natural and Synthetic Visual Speech

#### Participants

Twelve volunteers (8 female, 11 right-handed), mean age 35 years (range 22–47 years), participated in Experiment 1. Participants were recruited from an existing database of volunteers screened to have normal or corrected-to-normal vision, normal hearing, and lipreading ability no worse than one-half standard deviation below the mean for hearing individuals on a sentence lipreading screening test (Auer and Bernstein, 2007). There are large individual differences in lipreading ability among adults with normal hearing (Bernstein et al., 2000; Auer and Bernstein, 2007); poor lipreaders were excluded as they might generate little useful data. All participants gave informed consent and were compensated financially for their participation. Most participants completed the experiment in fewer than 3 h. The research was approved by the University of Southern California and St. Vincent’s Hospital (Los Angeles, CA, USA) Institutional Review Boards for the use of human subjects.

#### Stimuli

Natural CV syllables were recorded with a production quality camera (Sony DXC-D30 digital) and video recorder (Sony UVW 1800). Simultaneously, retro-reflectors on the talkers face were recorded using a three-camera, three-dimensional motion capture system (Qualisys MCU120/240 Hz CCD Imager). **Figure 1A** shows the retro-reflector positions, which included three on the forehead that were used to separate and eliminate overall head movement from visible speech movement in the synthesized video stimuli. The speech stimuli were from recordings of Talker M2 in (Jiang et al., 2007), from which tokens were selected that were free of large head motion and noticeable artifacts such as large eye movements and non-verbal mouth motion. The syllables used in this study were /bɑ, pɑ, dɑ, tɑ, gɑ, kɑ, dƷɑ, tʃɑ, Ʒɑ, hɑ, lɑ, nɑ, rɑ, vɑ, fɑ/.

The stimulus selection used for the discrimination testing is described in detail in **Table 1**. The table is organized in terms of stimulus pair triplets, for which each anchor stimulus is part of a (1) a *same* stimulus pair (i.e., *anchor* vs. *anchor*) but using different tokens, (2) a *far* pair with a large physical distance (i.e., *anchor* vs. *far*), and (3) a *near* pair with an intermediate physical distance (i.e., *anchor* vs. *near*). Same pairs used different recorded tokens so that speech-related differences that were not phonemic would be present in the stimulus set, in addition to non-speech token differences. PEC groupings that were computed across all of the initial consonants in CV syllables were /w, r/, /m, p, b/, /f, v/, /Θ, ð/, /t, d, s, z, ʃ, Ʒ, ʧ, dƷ/ and /y, l, n, k, h, g/ (Jiang et al., 2007). Thus, *far* pairs were from across PECs (visemes), and *near* pairs were from within PECs. *Same* pairs used different stimulus tokens in order to defend against discrimination judgments based on irrelevant (non-speech) stimulus attributes such as the talker’s eye gaze. Different pairs selected from two tokens of each syllable, so that discrimination between phonemes generalized across tokens. In Jiang et al. (2007), the largest physical stimulus distances for this speech were ∼4, and here *far* pairs had distance of ∼4. *Near* pairs corresponded to physical stimulus distances of ∼2.

**Table 1:** Summary of all stimulus pairs in Experiments 1 and 2.

Triplet anchor	Pair		Stimulus distance	Role
**Experiment 1**				
dɑ	pɑ	dɑ	3.92	*Far*
	pɑ	pɑ	0	*foil (same)*
	Ʒɑ	dɑ	2.32	*Near*
	dɑ	dɑ	0	*Same*
dƷɑ	vɑ	dƷɑ	3.96	*Far*
	vɑ	vɑ	0	*foil (same)*
	dɑ	dƷɑ	2.88	*Near*
	dƷɑ	dƷɑ	0	*Same*
kɑ	rɑ	kɑ	4.08	*Far*
	rɑ	rɑ	0	*foil (same)*
	hɑ	kɑ	2.27	*Near*
	hɑ	hɑ	0	*foil (same)*
	kɑ	kɑ	0	*Same*
lɑ	ʧɑ	lɑ	4.04	*Far*
	ʧɑ	ʧɑ	0	*foil (same)*
	gɑ	lɑ	1.96	*Near*
	gɑ	gɑ	0	*foil (same)*
	lɑ	lɑ	0	*Same*
	lɑ	kɑ	2.05	*foil (different)*
nɑ	vɑ	nɑ	4.24	*Far*
	kɑ	nɑ	2.15	*Near*
	nɑ	nɑ	0	*Same*
	nɑ	kɑ	2.15	*foil (different)*
tɑ	bɑ	tɑ	3.99	*Far*
	bɑ	bɑ	0	*foil (same)*
	dƷɑ	tɑ	2.28	*Near*
	tɑ	tɑ	0	*Same*
Ʒɑ	fɑ	Ʒɑ	4.04	*Far*
	fɑ	fɑ	0	*foil (same)*
	tɑ	Ʒɑ	2.28	*Near*
	Ʒɑ	Ʒɑ	0	*Same*
**Experiment 2**				
dɑ	pɑ	dɑ	3.92	*Far*
	pɑ	pɑ	0	*Foil (same)*
	Ʒɑ	dɑ	2.32	*Near*
	Ʒɑ	Ʒɑ	0	*Foil (same)*
	dɑ	dɑ	0	*Same*
dƷɑ	vɑ	dƷɑ	3.96	*Far*
	vɑ	vɑ	0	*foil (same)*
	dɑ	dƷɑ	2.88	*Near*
	dƷɑ	dƷɑ	0	*Same*
	dƷɑ	tɑ	2.28	*Foil (different)*
kɑ	rɑ	kɑ	4.08	*Far*
	rɑ	rɑ	0	*foil (same)*
	hɑ	kɑ	2.27	*Near*
	hɑ	hɑ	0	*Foil (same)*
	kɑ	kɑ	0	*Same*
nɑ	vɑ	nɑ	4.24	*Far*
	kɑ	nɑ	2.15	*Near*
	nɑ	nɑ	0	*Same*
	nɑ	kɑ	2.15	*Foil (different)*

*Stimuli were organized in terms of triplets of *same, far*, and *near* stimuli relative to an anchor. Foil stimuli were selected to defeat discrimination judgments based on stimulus position in a discrimination trial. Foils may serve that purpose for more than one triplet in the table. Stimulus distance is the perceptually warped stimulus distance from Jiang et al. (2007). Experiment 2 stimuli were a subset of those in Experiment 2.*

The stimulus generation organized in terms of sets of triplets afforded collection of valid response time measures to evaluate perception. The mean syllable duration was 0.530 s. But the range was 0.270–1.120 s. In order to compare latencies across pair distance, it was necessary to present anchors second in each trial, measure response times from the anchor’s onset, and compare latencies within triplets. This approach had the side effect, however, of requiring foil stimulus pairs, so that stimulus position could not be used as a clue to the correct discrimination response (see **Table 1**). *Same* foils were added to the stimulus set whenever the *near* or *far* phoneme in a triplet never served as an anchor in another triplet. *Different* foils were added whenever an anchor in one triplet did not appear in any other triplet: Otherwise, whenever it was first in a trial the correct response *same* would be obvious. Some foil trials served that purpose for more than one triplet.

All stimuli were edited so that the first video frame showed the mouth in closed position and the moving stimulus ended when the mouth reached maximal opening and jaw drop. Thus, the stimulus was truncated to reduce testing time, without sacrificing relevant stimulus information. Pilot testing suggested that this truncation scheme did not affect discrimination accuracy. The initial video frame (at 29.97 frames/s) of the still face was repeated five times before the speech stimulus began to move, and the final frame was repeated five times at the end, resulting in ten frames. The inter-stimulus-interval for each trial was 333 ms of neutral face.

#### Synthetic Stimuli

Visual speech stimuli were synthesized based on the three-dimensional optical data that were recorded simultaneously with the video. The synthetic talker (Jiang et al., 2008) (**Figure 1B**) was based on a wire frame mesh of three-dimensional polygons that defined the head and its parts (Xue et al., 2006). The original three-dimensional face model was obtained from www.digimation.com. The model was later edited (addition, deletion, and modification of some vertices, polygons, and textures) to have 1915 vertices and 1944 polygons. An algorithmic layer allowed the mesh to be deformed for performing facial actions as well as preventing errors (such as incursion of the lower lip into the volume of the upper lip). Optical trajectories were registered (calibrated) onto the key points on the face model, and these key points were used to deform the rest of the face vertices using a modified radial basis functions (Ma et al., 2006; Xue et al., 2006). Reconstructed 3-D motion data were processed to remove head motion, compensate for missing data and for eyebrow motion, remove noise, normalize the head-size, and smooth the motion tracks. Texture was re-mapped onto the deformed face and animation with lighting and background using the *openGL* graphics application-programming interface. Lighting in the face animation was chosen to be close to that of the natural video. The synthetic face was scaled and shifted to have the same position and size as the natural face (see **Figures 1A,B**). The animation had a resolution of 720 × 480 pixels. The resulting 60-Hz AVI videos were then interlaced to produce 30-Hz video. Using this model previously (Jiang et al., 2008), synthesized versus natural words were presented in pairs for discrimination, and perceptual distance was varied among word pairs. The results showed that perceivers could judge across the natural versus synthetic stimuli whether the words were the *same* or *different*, suggesting that the synthesis generated perceptually useful speech information.

#### Procedures

Natural and synthetic CV stimuli were presented on a CRT monitor. The video source was a DVD player driven by custom software. The discrimination testing was followed by a forced choice perceptual identification test, with the order of natural versus synthetic stimuli during testing the same across the discrimination and identification paradigms but counter-balanced across participants.

Prior to discrimination testing, participants were told that they would see video clips of a face silently speaking pairs of nonsense syllables, and that their task was to judge if the two syllables were the *same* or *different*. Instructions emphasized that even when the syllables were the same, the two video clips would be different. Participants were instructed to respond as quickly and as accurately as possible. Two brief six-trial practice blocks—one with natural stimuli, the other with synthetic—preceded the experimental data collection.

Each of the 94 stimulus pairs comprising a discrimination block was presented in a pseudo-random order. Blocks were repeated 10 times per stimulus type (synthetic and natural) with a new pseudo-random order on each block. The hand used to report *same* or *different* was counter-balanced across participants. Feedback indicating the correct response was delivered via a pair of blue light-emitting diodes mounted on the sides of the video display. Response times were recorded using a custom-built timer, triggered by a specially designed audio track on the DVD, which was verified to ensure accurate and reliable synchronization between the video stimulus and the response timer.

Following the discrimination task, closed-set perceptual identification was carried out on the 15 syllables (two tokens each) from the triplet stimulus sets in the discrimination task. Stimuli were presented singly in blocks of natural or synthetic stimuli. Participants identified each CV syllable by using a computer mouse to click one of 15 labeled response buttons on a separate display. Each button showed a letter and an example word to identify the phoneme. Stimuli were presented ten times each blocked by natural or synthetic type. No feedback was provided, and response times were not recorded. Two brief six-trial practice blocks—one with natural stimuli, the other with synthetic—preceded the experimental data collection.

#### Analyses

Statistical analyses were carried out using SPSS version 17.0 and MATLAB 7.10. Analysis was primarily repeated-measures analysis of variance (ANOVA) with degrees of freedom corrected for violations of sphericity using the Huynh-Feldt correction (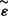) when needed. To provide a measure of effect size, η^2^ values are reported. Error bars in figures are within-subjects 95% confidence intervals (Morey, 2008).

#### Discrimination Data

Analyses on discrimination data were limited to triplet set stimulus pairs and not foil pairs. Percent correct was calculated as the proportion of *different* responses for *near* and *far* trials, and *same* responses for *same* trials. Discrimination sensitivity, *d’*, was calculated on a per-triplet basis using the common normal and equal-variance assumption. Within a triplet, the *false alarm* rate was the proportion of times the *different* response was given when the syllables were the same, and the *correct detection* rate was the proportion of times the *different* response was given when the syllables were different (either *near* or *far)*. *d*’ was computed as [Z(correct detection) - Z(*false alarm*)]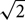, where *Z*() is the inverse cumulative normal distribution function. The multiplication by 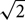 is used for a roving standard paradigm and transforms the resulting *d’* into an estimate of the sensitivity to the difference between the two syllables in a *near* or *far* trial (Macmillan and Creelman, 1991, pp. 155–158).

#### Response Time Data

Discrimination response times on correct (non-foil) trials were analyzed per participant. Outliers were removed by calculating the participant’s response time mean and SD and using only those response times within 2.5 times of their SD. Fewer than 3% of trials were excluded by this approach.

#### Identification data

Percent correct was calculated per participant and consonant. Analyses involving proportion correct measures were carried out with and without applying the arcsine transformation to stabilize variance. All of the results were replicated across transformed and untransformed scores. For simplicity and ease of interpretation, the proportion correct results are presented rather than the transformed score results.

Shannon entropy (Shannon, 1948) was calculated for each syllable as -∑ _n_plog_2_p, where *n* is the number of response categories (i.e., initial consonants in syllables), and *p* is the proportion of responses in that category. Low entropy implies that responses to the stimulus were assigned to one or a small number of syllables. A high value implies that responses are distributed across available response categories. With the 15 alternatives in the identification task, entropy ranges between 0 for all correct responses or use of one incorrect response, to 3.9 for an equal number of responses in each cell of the confusion matrix.

### Results

#### Discrimination

**FIGURE 2:**
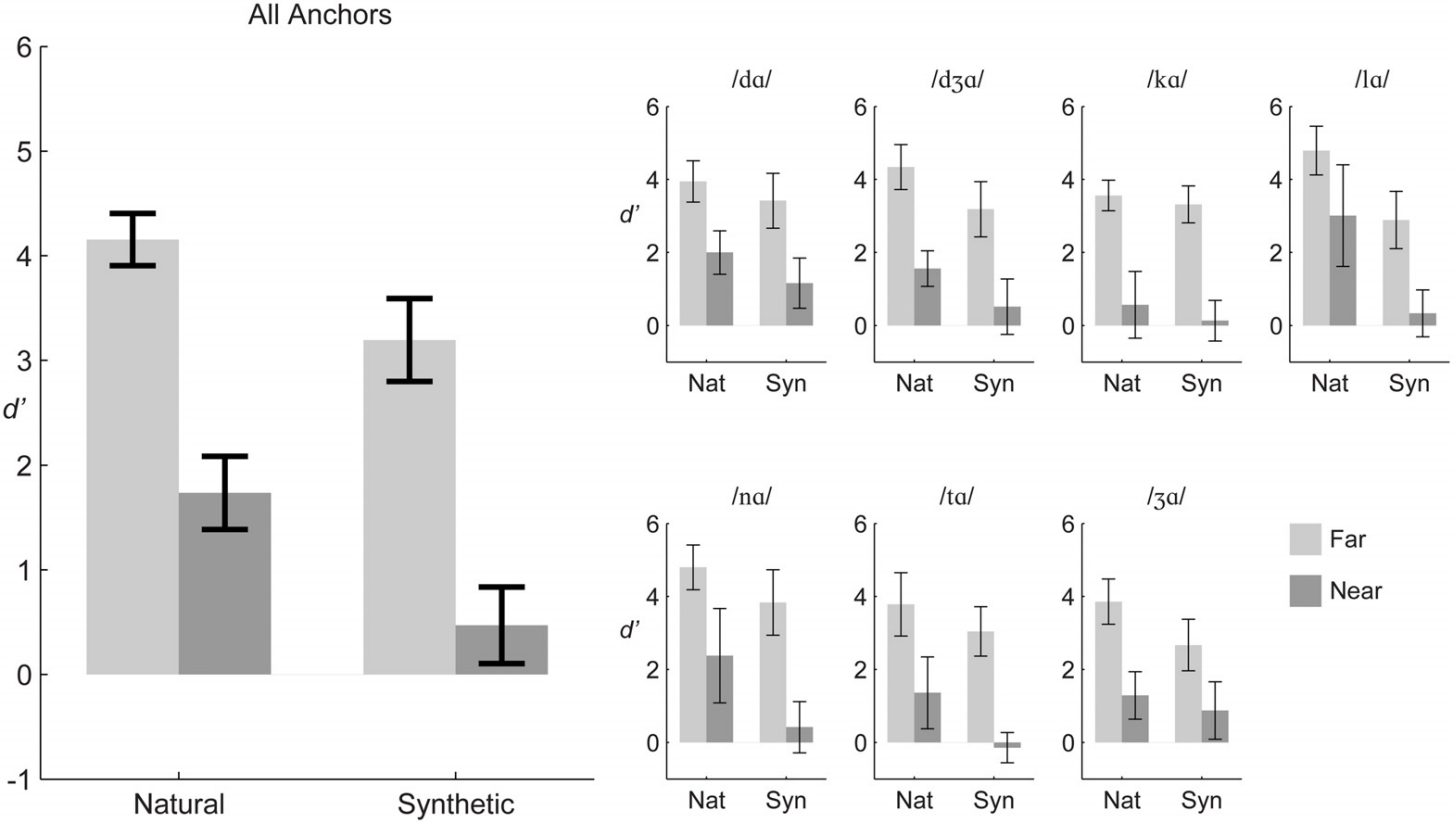
**Experiment 1 group mean *d’* sensitivity.** The large panel **(Left)** shows results averaged across stimulus triplets, and the small panels **(Right)** show results for each triplet. Error bars are within-subjects 95% confidence intervals.

**Figure 2** shows that group mean *d’* scores for natural and synthetic stimulus pairs were higher for *far* pairs compared to *near* pairs collapsed over the different syllable triplets. The figure shows that the same pattern held when syllable triplets were considered separately.

Repeated-measures ANOVA was carried out on *d’* scores with the within-subject factors of distance (*far, near*), stimulus type (natural, synthetic), and triplet (7). The distance and stimulus type main effects were reliable. *Far* pairs were discriminated more accurately than *near* pairs, *F*(1,11) = 337.5, η*^2^* = 0.573, *p* < 0.001 (*far mean d’* = 3.67, *near mean d’* = 1.10). Natural stimuli were discriminated more accurately than synthetic stimuli, *F*(1,11) = 65.9, η*^2^* = 0.108, *p* < 0.001 (natural *mean d’* = 2.94, synthetic *mean d’* = 1.83).

However, there were reliable two-way and three-way interactions among distance, stimulus type, and triplet. In order to gain insight into the interactions, repeated measures ANOVA was carried out for each of the seven triplets. The within-subjects factors were distance (*far, near*) and stimulus type (natural, synthetic). Statistics for each triplet are given in **Table 2**. Across triplets, the main effect of distance, with *d’* for *far* pairs higher than for *near* pairs, and the main effect of stimulus type, with *d’* for natural higher than *d’* for synthetic pairs, were reliable. Interactions of distance with stimulus type were reliable for the anchors /nɑ/, /tɑ/, and /Ʒɑ/. For triplets with anchor syllables /nɑ/ and /tɑ/, the interaction was attributable to a larger effect of stimulus type for the *near* pairs than the *far* pairs. For the triplet with anchor syllable /Ʒɑ/ the interaction was attributable to a smaller effect of stimulus type for the *near* pair than the *far* pair.

**Table 2:** Analyses of variance on *d’* for the triplets in Experiment 1.

Anchor	Source	*df*	*F*	η^2^	*p*	Mean difference	Confidence interval
/dɑ/	Distance (*far, near*)	1	364.3	0.77	0.001	2.10	[1.86 2.35]
	Type (natural, synthetic)	1	9.7	0.08	0.010	0.69	[0.20 1.17]
	Distance with type	1	1.62	0.00	0.230		
	Error	11		0.14			
/dƷɑ/	Distance (*far, near*)	1	220.6	0.74	0.000	2.73	[2.32 3.13]
	Type (natural, synthetic)	1	20.2	0.12	0.001	1.10	[0.56 1.64]
	Distance with type	1	0.1	0.00	0.770		
	Error	11		0.14			
/kɑ/	Distance (*far, near*)	1	217.6	0.89	0.000	3.09	[2.63 3.55]
	Type (natural, synthetic)	1	4.4	0.01	0.059	0.34	[-0.02 0.69]
	Distance with type	1	0.4	0.00	0.530		
	Error	11		0.09			
/lɑ/	Distance (*far, near*)	1	32.4	0.36	0.000	2.17	[1.33 3.01]
	Type (natural, synthetic)	1	51.6	0.40	0.000	2.29	[1.59 3.00]
	Distance with type	1	0.4	0.01	0.530		
	Error	11		0.24			
/nɑ/	Distance (*far, near*)	1	78.7	0.60	0.000	2.92	[2.19 3.64]
	Type (natural, synthetic)	1	25.6	0.15	0.003	1.46	[0.60 2.33]
	Distance with type	1	6.0	0.02	0.032		
	Error	11		0.23			
/tɑ/	Distance (*far, near*)	1	217.7	0.74	0.000	2.80	[2.39 3.22]
	Type (natural, synthetic)	1	18.4	0.12	0.001	1.12	[0.55 1.70]
	Distance with type	1	10.1	0.01	0.009		
	Error	11		0.12			
/Ʒɑ/	Distance (*far, near*)	1	156.6	0.70	0.000	2.18	[1.80 2.56]
	Type (natural, synthetic)	1	12.0	0.10	0.005	0.80	[0.29 1.31]
	Distance with type	1	5.7	0.02	0.035		
	Error	11		0.18			

*Each of the anchor stimuli and its triplet set were analyzed separately (see text). Mean difference is level 1 minus level 2 (i.e., far minus near, natural minus synthetic). Values in brackets are lower and upper 95% confidence intervals.*

To assess whether discrimination was reliably different from zero, one-sample *t*-tests (*df* = 11) were run for each stimulus pair. After Bonferroni correction, all *far* pairs (natural and synthetic) were reliably discriminable, as were all natural *near* pairs with the exception of /hɑ/-/kɑ/. These results support the expectation that discrimination is possible within the same visemes for natural speech. For synthetic speech only two of the synthesized near pairs were reliably discriminable: /tɑ/-/Ʒɑ/ and /Ʒɑ/-/dɑ/.

#### Discrimination Response Times

**FIGURE 3:**
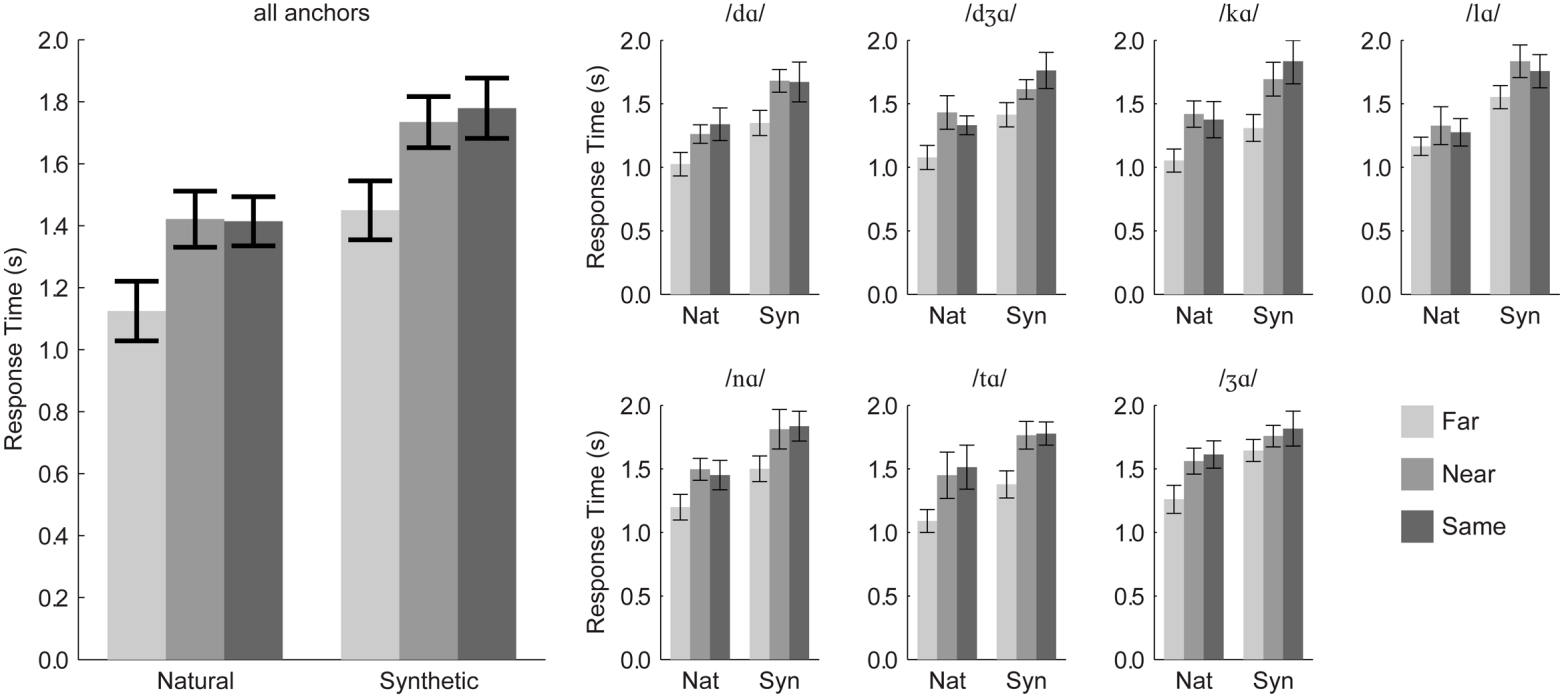
**Experiment 1 response times.** Group mean response times pooled over anchor syllable are shown in the **(Left)**, and group mean response times for each anchor syllable are shown in the **(Right)**. Error bars are within-subjects 95% confidence intervals.

After removing outliers, 97.3% of the 12,545 response times were retained. Individual responses times were generally long (*mean =* 1,231–1,781 ms), because measurements were initiated at the beginning of the triplet stimulus in the second trial interval. Results are summarized in **Figure 3**.

Response times were submitted to a repeated-measures ANOVA with the within-subjects factors of distance category (*same, near, far*), stimulus type (natural, synthetic), and triplet. (One participant’s data were excluded, because they could not discriminate /lɑ/-/ gɑ/ with the synthetic visible speech). There was a significant main effect of distance, *F*(2,20) = 38.9, η*^2^* = 0.345, *p* < 0.001. *Far* pair responses (mean *RT* = 1,293 ms) were faster than *near* (mean *RT* = 1,592 ms) and *same* (mean *RT* = 1,628 ms) pairs. *Far* syllables were discriminated more than 300 ms faster than *near* or *same* syllables. Thus, there was no speed/accuracy trade-off in this task. Stimulus type was a reliable factor, *F*(1,10) = 282.6, η*^2^* = 0.490, *p* < 0.001, with responses to natural stimulus pairs (mean *RT* = 1,326 ms) being faster than those to synthetic stimulus pairs (mean *RT* = 1,683 ms).

Order (natural first, synthetic first) was used in this analysis. It was not a reliable main effect, *F*(1,9) = 0.3, η*^2^* = 0.024, *p* = 0.624, but it interacted with stimulus type, *F*(1,9) = 19.2, η*^2^* = 0.020, *p* = 0.002. Both presentation orders resulted in reliably faster responses to natural compared to synthetic stimuli, but the response time disadvantage of synthetic stimuli was smaller for participants who discriminated natural stimuli first.

Because there were interactions with the anchor factor, repeated-measures ANOVA was applied separately for each triplet (7) with within-subjects factors of distance category (*far, near, same*) and stimulus type (*natural, synthetic*), and between-subjects factor of presentation order (*natural* first, *synthetic* first). Statistics are reported in **Table 3**. All of the main effects of distance category and stimulus type were reliable. Responses to natural stimuli were always faster than responses to synthetic stimuli, and *far* pair responses were always faster than *near* or *same* pair responses.

**Table 3:** Analyses of variance for response times in Experiment 1.

Anchor	Source	*df*	*F*	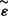	η*^2^*	*p*
/dɑ/	Distance (*far, near, same*)	2, 20	17.8	1	0.29	0.000
	Type (natural, synthetic)	1, 10	198.2	1	0.46	0.000
	Distance with type	2, 20	1.6	1	0.01	0.230
/dƷɑ/	Distance (*far, near, same*)	2, 20	23.8	1	0.30	0.000
	Type (natural, synthetic)	1, 10	348.7	1	0.41	0.000
	Distance with type	1.6, 16.6	7.2	0.83	0.04	0.004
/kɑ/	Distance (*far, near, same*)	2, 20	17.8	1	0.41	0.000
	Type (natural, synthetic)	1, 10	129.2	1	0.31	0.000
	Distance with type	1.4, 14.2	3.9	0.71	0.02	0.055
/lɑ/	Distance (*far, near, same*)	1.9, 17.4	17.8	0.97	0.11	0.000
	Type (natural, synthetic)	1, 9	88.6	1	0.65	0.000
	Distance with type	2, 18	0.9	1	0.01	0.436
/nɑ/	Distance (*far, near, same*)	2, 20	23.0	1	0.30	0.000
	Type (natural, synthetic)	1, 10	80.4	1	0.42	0.000
	Distance with type	2, 20	0.8	1	0.00	0.471
/tɑ/	Distance (*far, near, same*)	2, 20	31.8	1	0.43	0.000
	Type (natural, synthetic)	1, 10	70.9	1	0.26	0.000
	Distance with type	1.4, 14.2	0.1	0.71	0.00	0.819
/Ʒɑ/	Distance (*far, near, same*)	2, 20	17.3	1	0.27	0.000
	Type (natural, synthetic)	1, 10	147.9	1	0.36	0.000
	Distance with type	2, 20	6.3	1	0.04	0.008

*Each of the anchor stimuli and its triplet set were analyzed separately (see text).*

#### Correlations between *d’* and Modeled Perceptual Dissimilarity

**FIGURE 4:**
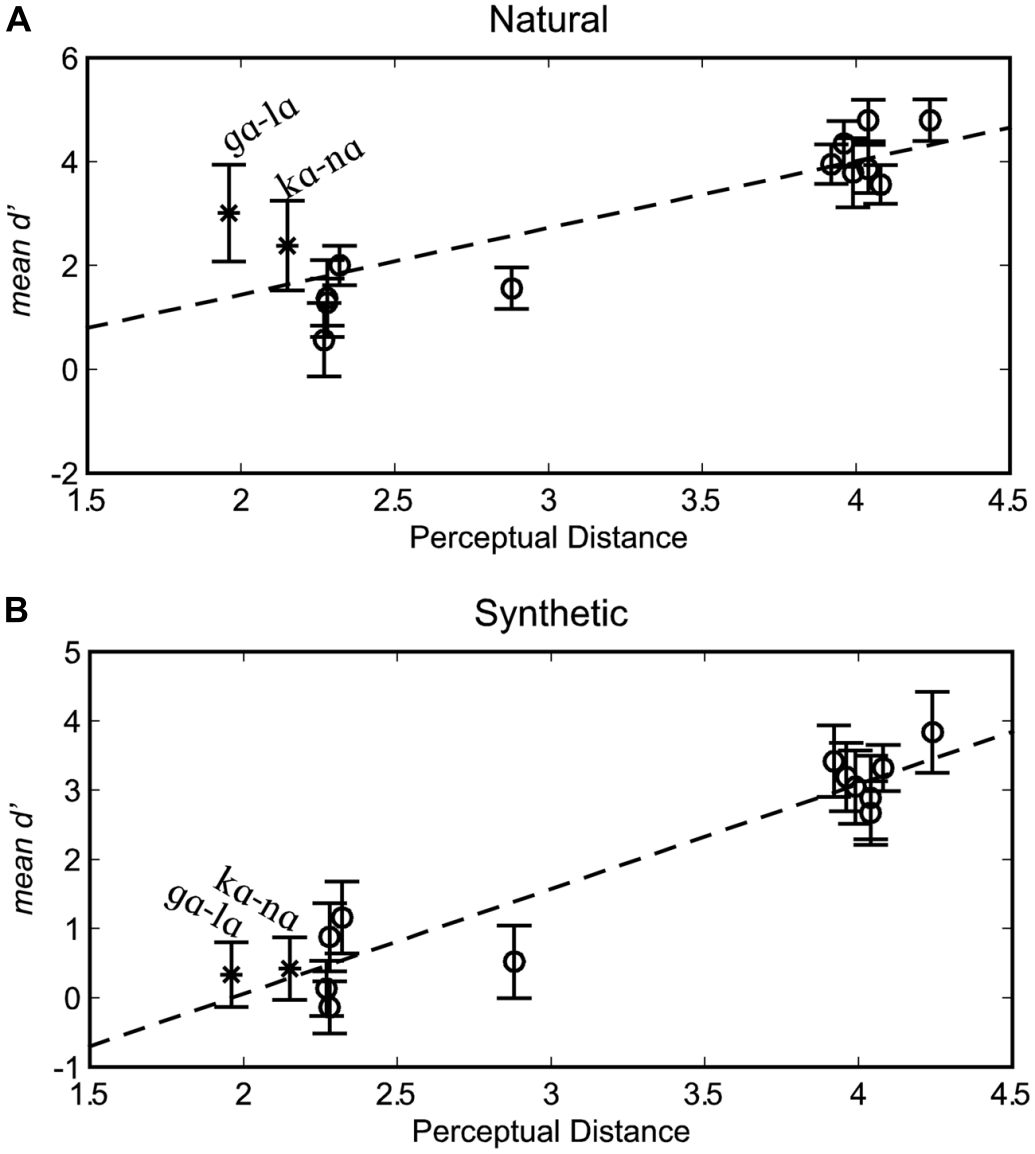
**Correlations between *d’* scores and perceptually warped physical distance.** Results are shown separately for natural **(A)** and synthetic **(B)** syllable pairs. Error bars are within-subjects 95% confidence intervals.

The organization of stimuli into triplet sets converted continuous perceptually warped physical measures from Jiang et al. (2007) into distance categories. In order to test the association between continuous distance and *d’*, the modeled perceptual distances from Jiang et al. (2007) were used in computing Pearson correlation coefficients (**Figure 4**), with each participant contributing a score for each stimulus pair. The correlation between the perceptually warped physical dissimilarity and *d’* for natural stimuli was *r*(166) = 0.676, *p* < 0.001, and the correlation for synthetic stimuli was *r*(166) = 0.828, *p* < 0.001.

Pearson correlations were also computed separately for each participant. For natural stimuli, the correlations between *d’* and perceptually warped physical distance were statistically reliable (*p <* 0.05) in 10 out of the 12 participants, *mean r*(12) = 0.720, *range r*(12) = 0.446–0.893 (variance accounted for range 19.9–79.7%). For synthetic stimuli, the correlations were reliable for all 12 participants, mean *r*(12) = 0.861, range *r*(12) = 0.710–0.981 (variance accounted for range 50.4–96.2%). Correlation coefficients were reliably higher for synthetic compared to natural visible speech, as tested using a paired-samples permutation test (*p* = 0.004). The scatter plot for perceptual distance versus *d*’ sensitivity (**Figure 4**) suggests that stimulus pairs, /lɑ/-/gɑ/ and /nɑ/-/kɑ/ were responsible for the difference in correlations across natural versus synthetic stimuli.

#### Identification

##### Percent correct

**FIGURE 5:**
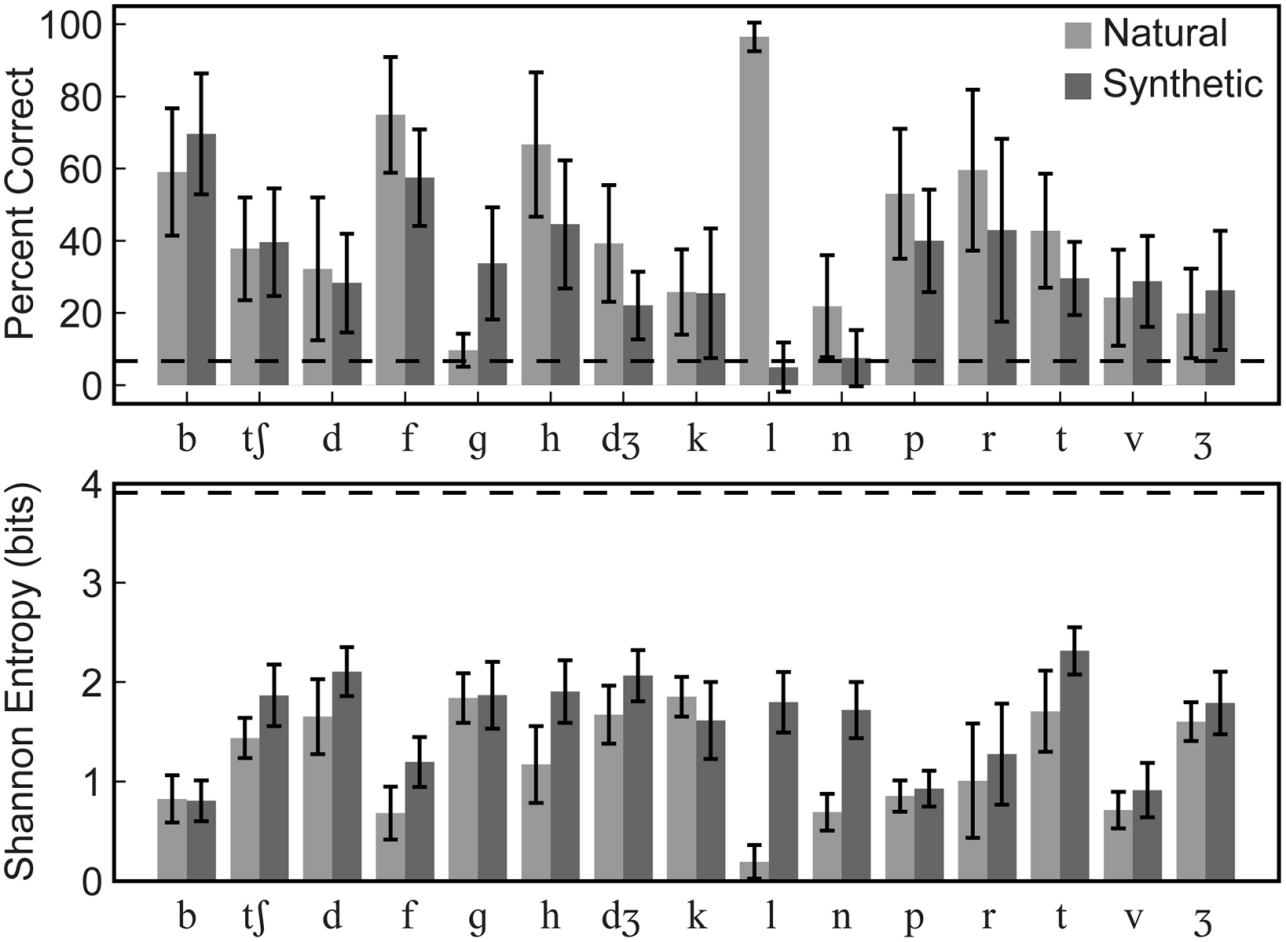
**Experiment 1 group mean phoneme identification percent correct and entropy.** Group mean percent correct **(upper)** and Shannon entropy **(lower)** are shown. Error bars show within-subjects 95% confidence intervals. Correct identification was reliably above chance (6.7%, dashed line) for all syllables except /gɑ/ in the natural type and /lɑ/ and /nɑ/ in the synthetic type. Even for cases with low percent correct identification, entropy was generally low, well below the theoretical maximum for this task (3.91, dashed line), indicating that responses were typically allocated to a small number of response categories.

**FIGURE 6:**
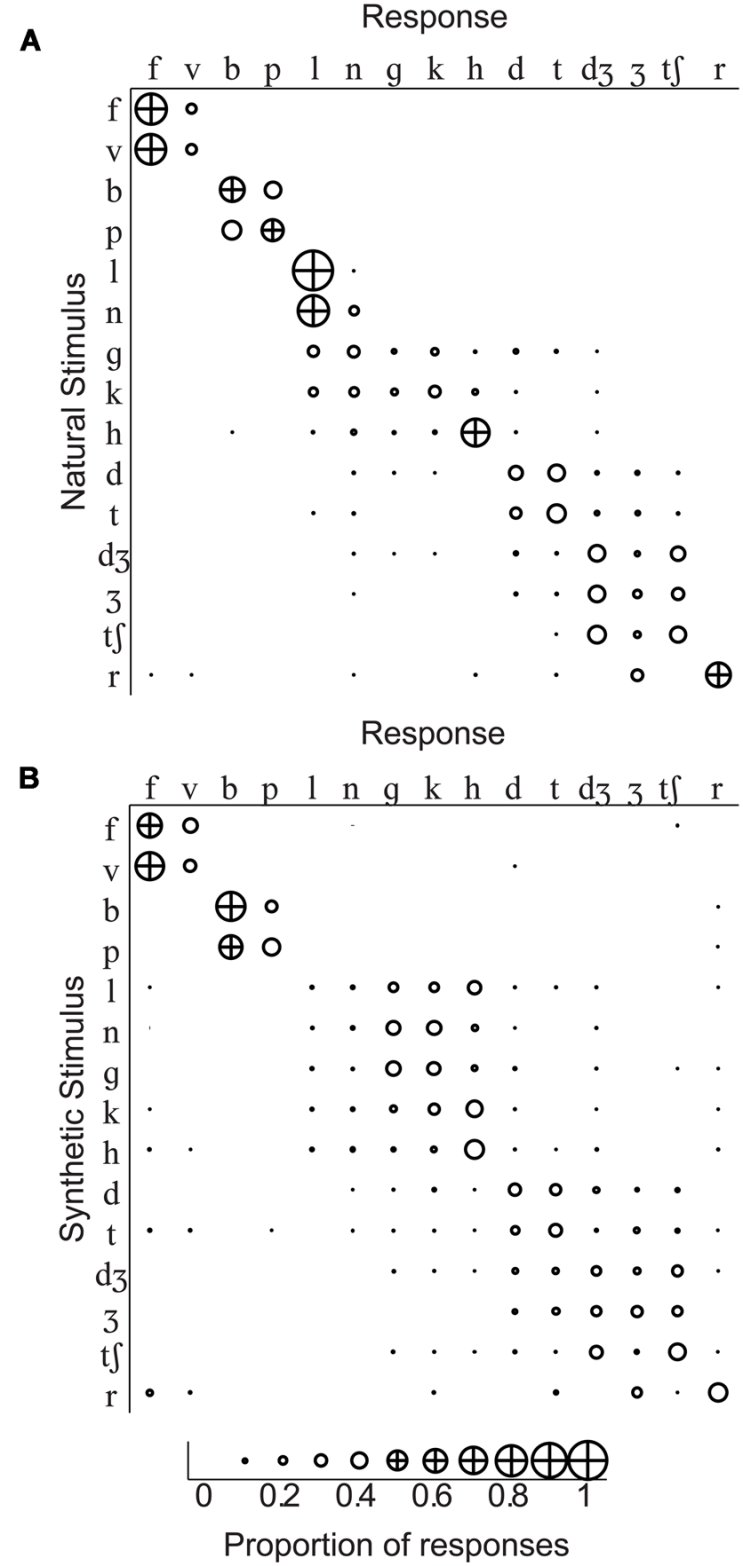
**Identification confusion matrices.** Group response proportions are shown for identification of **(A)** natural stimuli and **(B)** synthetic stimuli. The initial consonant of the CV stimulus (with /a/ in the vowel context) is shown at the head of each row, and responses to that stimulus are in separate columns. Correct responses fall on the diagonal, and incorrect responses are in off-diagonal cells of the matrix.

Group mean percent correct phoneme identification is shown in the upper panel of **Figure 5**. No individual participant’s 95% confidence interval computed using the binomial distribution included the percent correct expected by chance, 6.67% for either natural or synthetic speech. The scores are similar to those obtained in Jiang et al. (2007) for the natural speech of Talker M2 in the /ɑ/ context. The range of correct scores was 35–45%. Stimulus-response confusion matrices are shown in **Figure 6**.

A repeated measures ANOVA for percent correct consonants was carried out with the within-subjects factors of stimulus type (natural, synthetic), and consonant /bɑ/, /tʃɑ/, /dɑ/, /fɑ/, /gɑ/, /hɑ/, /dƷɑ/, /kɑ/, /lɑ/, /nɑ/, /pɑ/, /rɑ/, /tɑ/, /vɑ/, and /Ʒɑ/. Natural video speech was perceived more accurately (mean percent correct = 44.2) than synthetic (mean percent correct = 33.4), *F*(1,11) = 96.2, η*^2^* = 0.032, *p* < 0.001. However, the small eta squared shows that the effect was small. Consonant was also a reliable factor, *F*(12.2,134.6) = 6.91, η*^2^* = 0.264, *p* < 0.001, as was its interaction with stimulus type, *F*(10.9,120.0) = 16.52, η*^2^* = 0.169, *p* < 0.001. Follow-up paired *t*-tests showed that natural visible speech consonants /fɑ, hɑ, dƷɑ, lɑ, nɑ, rɑ/ were more accurately identified than their synthetic counterparts, and synthetic /gɑ/ was more accurately identified than its natural counterpart, *p* < 0.05 (uncorrected).

##### Entropy

Group mean Shannon entropy is shown in the lower panel of **Figure 5**. A repeated-measures ANOVA was carried out on entropies with within-subjects factors of stimulus type (natural, synthetic) and consonant (/bɑ/, /tʃɑ/, /dɑ/, /fɑ/, /gɑ/, /hɑ/, /dƷɑ/, /kɑ/, /lɑ/, /nɑ/, /pɑ/, /rɑ/, /tɑ/, /vɑ/, /Ʒɑ/) and between-subjects factor of presentation order (natural first, synthetic first). Lower entropy was obtained with natural than with synthetic stimuli, *F*(1,10) = 77.3, η*^2^* = 0.092, *p* < 0.001 (natural, mean entropy = 1.19; synthetic, mean entropy = 1.61). But consonant was a reliable factor, *F*(7.9,79.2) = 15.7, η*^2^* = 0.385, *p* < 0.001, and there was an interaction between consonant and stimulus type, *F*(12.5,124.7) = 8.1, η^2^ = 0.104, *p* < 0.001. Follow-up paired comparisons showed that there was significantly lower entropy for the natural compared to synthetic /fɑ, hɑ, lɑ, nɑ, tʃɑ, dɑ, tɑ/ stimuli, *p* < 0.05 (uncorrected).

#### Discussion

Experiment 1 showed that natural and synthetic visual speech consonants from between and within PEC (viseme) groups were discriminable, and that discriminability was associated with perceptually warped physical stimulus dissimilarity. Discriminability was also inversely associated with response times, the more discriminable the stimuli, the shorter the response latency. Natural speech was more discriminable and resulted in shorter latencies than synthesized speech. Natural and synthetized stimuli were identified at above chance levels, but natural stimuli were more accurately identified. Errors were more systematic with natural speech. These results are compatible with the evidence summarized in Section “Introduction” that suggests that perceivers are sensitive to phoneme differences that are traditionally regarded to be invisible and non-informative.

The comparison between natural and synthesized speech supports the conclusion that lipreaders discern fine details in the natural visual stimulus. The synthetic stimuli did not incorporate a tongue model. It is likely that glimpses into the talker’s mouth are important for perception of place and manner of articulation. For example, articulation of /l/ involves a flattened tongue blade that can be glimpsed through the open lips when the consonant is followed by /ɑ/. Modeling of the lip motion using only the outer lip margin as was the case here reduces visible articulatory information, because the inner and outer lip margins are not necessarily correlated in their shape and movements. Also, the algorithmic approach to interpolating movement among sparse three-dimensional data such as for the synthetic talker’s cheeks probably reduces perceptible speech information. The overall implication of these effects is that perceivers are sensitive to and make use of the details in natural speech stimuli, and their sensitivity and response times are affected when those details are reduced or eliminated.

### Experiment 2: Inverted and Upright Visual Speech Discrimination

Several investigators have used inverted talking faces to evaluate aspects of audiovisual and visual-only speech perception (Massaro and Cohen, 1996; Rosenblum et al., 2000; Thomas and Jordan, 2004). In Massaro and Cohen (1996), inversion of four synthetic CV syllables reduced identification accuracy by ∼10% points. Among the four stimulus consonants, /b, v, d, ð/, /v/ was most affected by inversion. In Rosenblum et al. (2000), there were few identification errors to /b/ versus /v/. But errors in identifying /g/ were substantial in the contrast between /b/ and /g/. In Thomas and Jordan (2004), six CVC real words were presented for identification, and inversion reduced identification accuracy.

Face inversion reduces face identification accuracy, implying that internal face representations are not fully invariant to orientation (Yovel and Kanwisher, 2005; Jiang et al., 2006; Susilo et al., 2010; Gold et al., 2012). However, the Jiang et al. (2007) model that was used to generate dissimilarities for Experiment 1 did not take orientation into account. Its between-phoneme distance estimates also do not take into account the spatial organization of the individual motion points. In Experiment 2, a subset of the discrimination pairs from Experiment 1 was presented in order to probe whether speech discrimination is invariant to orientation. If perceivers were sensitive only to visual speech motion, as represented in the optical recordings, we would predict that orientation would not affect discrimination scores. This prediction is consistent with the organization of the visual pathways, which represent motion at a lower level of organization than complex multi-feature images, such as natural faces performing non-speech motions (Fox et al., 2009; Pitcher et al., 2011). If, however, perceivers use the spatial organization of talking face (i.e., their configuration), inversion is expected to diminish discrimination sensitivity and increase response times.

#### Methods

In Experiment 2, discrimination was carried out with upright versus inverted stimuli. No identification testing was carried out. Methods followed those from Experiment 1, except as described below.

##### Participants

Twelve volunteers (10 female, all right-handed, mean age 25 years, range 19–37 years), none from Experiment 1, gave written informed consent and were financially compensated for their participation.

##### Stimuli

Stimuli were four triplets from the natural stimuli in Experiment 1 (see **Table 1**) and only the foils needed in the context of the reduced set. The total number of stimulus pairs was 58; however, an additional pair, /gɑ/-/gɑ/ was inadvertently included, so the total number of pairs presented was 59. Stimuli were inverted by presenting them on an inverted monitor.

##### Procedure

Stimulus pairs were presented in pseudo-random order within a block. Blocks were repeated (with stimuli in a different order each time) a total of six times per condition (upright, inverted). All blocks of a particular condition were completed before blocks of the other condition were begun, with counter-balancing across participant groups. Before each condition there was a six-trial practice to familiarize the participant with the experimental setup. Because response time effects did not interact with button side mapping in Experiment 1, Experiment 2 did not counter-balance button side mapping.

### Results

#### Discrimination

**FIGURE 7:**
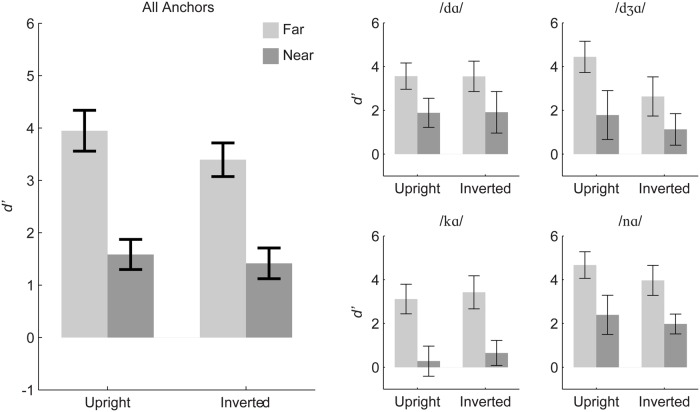
**Experiment 2 mean *d’* sensitivity for inverted and upright stimuli.** The left panel shows group mean d’ averaged over all anchors, and the small panels show group mean d’ separated out by triplet anchor. Error bars are 95% within-subjects confidence intervals.

**Figure 7** summarizes the discrimination results and suggests that the pattern of discrimination across *near* and *far* stimulus pairs was invariant to orientation. A repeated measures ANOVA was carried out with within-subjects factors of stimulus distance (*near, far*), orientation (upright, inverted), and anchor syllable (/dɑ/, /dƷɑ/, /kɑ/, /nɑ/). Distance was a reliable main effect, *F*(1,11) = 399.5, η^2^ = 0.525, *p* < 0.001, with *d’* for *far* (mean *d’* = 3.67) greater than *near* pairs (mean *d’* = 1.50). Anchor was a reliable main effect, *F*(2.13,23.43) = 11.21, ^∼^ 𝜀 = 0.710, η^2^ = 0.11, *p* < 0.001. However, orientation was also a reliable but very small main effect, *F*(1,11) = 4.85, η^2^ = 0.015, *p* = 0.05, with higher *d*’ for upright (mean *d’* = 2.77) than inverted stimulus pairs (mean *d’* = 2.41).

All of the interactions were reliable, but the effect sizes were small: Orientation with distance, *F*(1,11) = 7.15, η^2^ = 0.004, *p* = 0.022; Anchor consonant with distance, *F*(3,33) = 6.85, η^2^ = 0.019, *p* < 0.001; Orientation with anchor consonant, *F*(3,33) = 6.09, η^2^ = 0.04, *p* = 0.002; and Distance, orientation, and anchor, *F*(3,33) = 3.39, η^2^ = 0.006, *p* = 0.029.

Separate repeated measures ANOVAs were run for each of the four triplets with the factors orientation (upright, inverted) and stimulus distance (*near, far*) (see **Table 4**). The analyses suggest that the triplet with anchor /dƷɑ/ was the main source of effects involving orientation in the omnibus analysis, and within that triplet, the orientation effect was apparently due to the *far* pair. Paired *t*-tests revealed that there was a reliable inversion effect (upright more discriminable than inverted) for the *far* pair (/vɑ/-/dƷɑ/) but not for the *near* pair (/dɑ/-/dƷɑ/).

**Table 4:** Analyses of variance on *d’* for the triplets in Experiment 2.

Anchor	Source	*df*	*F*	η^2^	*p*	Mean difference	Confidence interval
/dɑ/	Distance (*far, near*)	1	399.5	0.64	0.001	1.66	[1.30 2.03]
	orientation (inverted, upright)	1	0.0	0.00	0.979	0.01	[-0.66 0.67]
	distance with orientation	1	0.0	0.00	0.905		
	Error	11		0.36			
/dƷɑ/	distance (*far, near*)	1	79.9	0.52	0.001	2.08	[1.57 2.59]
	orientation (inverted, upright)	1	13.7	0.18	0.003	-1.23	[-0.50 -2.00]
	distance with orientation	1	10.8	0.04	0.007		
	Error	11		0.26			
/kɑ/	distance (*far, near*)	1	257.0	0.86	0.001	2.80	[2.42 3.19]
	orientation (inverted, upright)	1	1.8	0.01	0.210	0.34	[-0.22 0.91]
	distance with orientation	1	0.1	0.00	0.773		
	Error	11		0.13			
/nɑ/	distance (*far, near*)	1	140.0	0.73	0.001	2.14	[1.74 2.53]
	orientation (inverted, upright)	1	4.2	0.05	0.065	-0.56	[-1.16 0.04]
	distance with orientation	1	1.3	0.00	0.275		
	Error	11		0.22			

*Each of the anchor stimuli and its triplet set were analyzed separately (see text). Mean difference is level 1 minus level 2 (i.e., *far* minus *near*, inverted minus upright). Values in brackets are lower and upper 95% confidence intervals.*

#### Response time

After removing outliers, 97.3% of the responses (4,396) were retained. A repeated-measures ANOVA was carried out with within-subjects factors of stimulus distance (*same, near, far*), orientation (upright, inverted), and anchor (/dɑ/, /dƷɑ/, /kɑ/, /nɑ/). The main effect of orientation was not reliable, *F*(1,10) = 0.5, η^2^ = 0.006, *p* = 0.495. Distance was a reliable main effect, *F*(2,20) = 31.1, η^2^ = 0.290, *p* < 0.001. Responses to *far* pairs (mean *RT* = 1,058) were faster than those to *near* (mean *RT* = 1,255) or *same* pairs (mean *RT* = 1,225), but *same* and *near* were not different.

Anchor was a reliable main effect, but this effect was largely attributable to differences in the duration of the anchor syllable. The interaction of distance category with anchor was reliable, and follow-up paired comparisons showed that all triplets exhibited the same pattern of difference observed in the main effect of distance category, namely that responses to *far* pairs were faster than to *near* or *same* pairs (all *p* < 0.05).

The interaction of stimulus orientation with distance category and anchor consonant accounted for a small but significant amount of the variance, *F*(3.3,32.7) = 2.8, η^2^ = 0.019, *p* = 0.049. Follow-up paired comparisons showed that in both upright and inverted conditions, the *far* pair response was faster than the *near* and *same* pair responses for all of the anchors, with the exception of the inverted *far* pair /vɑ/-/dƷɑ/ that was not faster than its *near* counterpart.

### Discussion

Experiment 2 showed that the effect size of stimulus inversion was small. The *d’* difference between upright and inverted stimuli was only 0.37. There was not a main effect of inversion on response time, although there were indications that inversion affected the perceived stimulus information in /vɑ/. There was reduction in discrimination sensitivity and no advantage in response time under inverted conditions for the /vɑ/-/dƷɑ/ *far* pair. Perception of the syllable /vɑ/ was previously shown to be affected by inversion (Massaro and Cohen, 1996; Rosenblum et al., 2000). In natural video, the /v/ is articulated with the teeth placed against the lower lip. This feature is highly visible. Thus, this result suggests that configural information is important at least for labiodental articulations, and that visual speech discrimination involves more than motion discrimination.

## General Discussion

This study shows that there is more phonetic information available in visual speech consonants than predicted by the notion of visemes as perceptual categories (Woodward and Barber, 1960; Fisher, 1968; Massaro et al., 2012). Experiment 1 showed that discrimination is excellent for consonants that represent different viseme-level PECs and is also reliable for consonant pairs within PECs. We therefore suggest that while the viseme, or more generally the PEC (Auer and Bernstein, 1997), can be useful for describing patterns of phoneme similarities and dissimilarities (Walden et al., 1977; Owens and Blazek, 1985; Iverson et al., 1998; Mattys et al., 2002) resulting from the invisibility of some articulatory information, the notion of the viseme as a perceptual category without internal perceptual structure or information is incorrect.

Experiment 1 also investigated the model by Jiang et al. (2007) which used consonant identification data and three-dimensional optical recordings to obtain perceptually warped physical stimulus distances. The experiment showed that discriminability is correlated with perceptually warped physical stimulus distance. The synthetic speech stimuli that were based on the three-dimensional optical recordings produced similar patterns of discriminability, although the synthetic speech was perceived less accurately. In addition, perceptual distances were predictive of response latencies. Thus, we have demonstrated complete consistency between earlier results on identification and distance from Jiang et al. (2007) and present results on discrimination and response latency. We comment further below on the potential usefulness of these relationships for determining the neural basis of visual speech perception.

Experiment 2 used stimulus inversion to probe whether visual speech information comprises solely motion attributes independent of orientation or face configuration. Some evidence was obtained concerning perception of /vɑ/ to support the expectation that face configuration is represented during visual speech perception, although the general pattern of better discriminability for *far* than for *near* stimuli held regardless of orientation. Coupled with the reduction in performance between natural and synthetic stimuli in Experiment 1, we conclude that visual speech perceivers are sensitive to relationships among face parts and not just to attributes of motion such as speed and velocity.

Our results on consonant identification in Experiment 1 once again demonstrated relatively low accuracy levels consistent with other reports in the literature. The range of natural consonant correct scores was 35–45%. In contrast to the view that low consonant identification invariably leads to the conclusion that lipreading must rely on higher-level linguistic and extra-linguistic knowledge, we again point out that partial phonetic information is regularly used to perceive speech as demonstrated by speech communication in noisy environments (Sumby and Pollack, 1954; MacLeod and Summerfield, 1987; Ross et al., 2007) and distorting environments (Grant et al., 2013) and by communication with hearing loss (Walden et al., 1975; Grant et al., 1998). Words can be identified even when they are phonetically impoverished, in part because the lexicon does not use all the phoneme combinations available within a language, and this is true even when the lexicon is modeled in terms of PECs (Auer and Bernstein, 1997; Iverson et al., 1998; MacEachern, 2000; Mattys et al., 2002; Feld and Sommers, 2011). The evidence here shows that the relatively poor phoneme identification was present in the same perceivers whose discrimination sensitivity extended to *near* stimulus pairs.

### Dissimilarity in Visual Speech Perception and Underlying Neural Representations

In addition to our interest here in discrimination results for demonstrating that perception is not limited to viseme categories, we are interested in how visual speech is represented by the brain. Recently, Bernstein and Liebenthal (2014) reviewed the perceptual evidence on visual speech in relationship to the neural evidence on the organization of the auditory and visual speech neural pathways. They presented a model of the auditory and visual speech pathways and their interactions during audiovisual speech perception. One of their strong suggestions was that the visual perception of speech relies on visual pathway representations of speech *qua* speech. That is, the visual system represents speech in parallel with the auditory system to at least the level of phonetic features or phonemes and possibly to the level of lexical forms. While we do not wish to reiterate their arguments here, we do want to discuss an example that demonstrates the importance of discrimination paradigms and measures of perceptual and physical stimulus dissimilarity for learning about how visual speech is represented by the brain.

A subset of the stimuli tested here was used in an EEG visual mismatch negativity (MMN; Pazo-Alvarez et al., 2003; Czigler, 2007; Kimura et al., 2011; Winkler and Czigler, 2012) study by Files et al. (2013). The visual speech syllables were selected to be *near* versus *far* in physical and perceptual distance (Jiang et al., 2007). Reliable visual MMN responses were observed in the higher-level visual pathway (posterior temporal cortex) on the left when the visual speech stimulus change was across *far* stimuli and in the homologous right hemisphere area to stimulus changes that were *far* and also *near*. The results were interpreted to be consistent with a specialization in the left posterior temporal cortex for phoneme category representation (Bernstein et al., 2011) and for sensitivity to phonetic change that may not be perceived as different phonemic categories on the right. Thus, the Files et al. (2013) study demonstrated the utility of knowledge about perceptual discrimination of visual speech for investigating the underlying neural representations of visual speech stimuli. In addition, their results are consistent with the findings reported here on *near* stimulus pair discrimination, that is that *near* differences are perceptible, although the finding that the right hemisphere is more sensitive to visual speech stimulus change raises interesting questions about its role in visual speech perception given the left-lateralization of speech processing in most right-handed individuals (Hickok and Poeppel, 2007; Desai et al., 2008; Rauschecker and Scott, 2009).

### Audiovisual Speech Processing

Many studies of audiovisual speech processing invoke the viseme as an established visual speech category in describing their stimulus selection, carrying out data analyses, and/or in theorizing (these references are examples of the typical usage: Campbell, 2008; Arnal et al., 2009; Kyle et al., 2013; Matchin et al., 2013; Jesse and McQueen, 2014)^2^. In many cases, the viseme concept may be a proxy for having measurements of phoneme identification and discrimination for the stimuli in the particular study. This practice may lead to incorrect inferences, not only because phonemes within visemes can be discriminated, but because visual phonemes vary in their phonetic information across talkers, stimulus tokens, and position in the syllable (Owens and Blazek, 1985; Demorest et al., 1996; Jiang and Bernstein, 2011; Auer and Bernstein, in preparation). Depending on the hypotheses being tested, using viseme categories may or may not be detrimental to the goals of the research.

The notion of the viseme may also be problematic for audiovisual neuroimaging research, because it may encourage simplification of what needs to be explained. Indeed, the neuroimaging research has focused on interaction or integration (Stein and Meredith, 1990) mechanisms rather than on the information that is integrated. Auditory speech is expected to be highly informative, but visual speech is not. If only a very small set of viseme categories can be perceived, one possibility is that the underlying neural representations of the talking face are not even specific to speech. This possibility seems consistent with the view that, “associations of speech processing with regions implicated in face processing suggest that seeing speech makes use of face-processing mechanisms” (p. 705) and, “some face processing appears to be a necessary, *if not a sufficient* [emphasis added], base for understanding speech from faces” (Campbell, 2011, p. p. 705). Viseme categories might simply correspond to motion features. This possibility seems consistent with the possibility that, “viseme-dependent facilitation” might depend on “visual motion but no detailed phonological information” (Arnal et al., 2009, p. 13445).

In fact, non-phonetic visual forms or motion, or even vibrotactile pulse-trains can interact with or influence auditory speech perception. For example, the onset of a rectangle or a tactile vibration can reduce uncertainty and improve speech detection (Bernstein et al., 2004; Tjan et al., 2014). A single uninformative lip gesture can improve forced choice identification of a small set of voiced versus unvoiced stimuli (Schwartz et al., 2004). Head motion correlated with the voice fundamental frequency can also improve auditory word identification in noise (Munhall et al., 2004). Audiovisual speech effects could arise in response to both phonetic and non-phonetic stimulus attributes.

As suggested earlier, this study supports the view that visual speech perception is not equivalent to either face motion or face configurations. Specifically, the synthetic stimuli were generated on the basis of sparse motion and configural data, and they were less well discriminated and identified than the video stimuli. This comparison strongly implies that visual speech perception relies on integration of both configural and motion features. In addition, Experiment 2 of the current study further demonstrated that orientation is a significant factor in visual speech discrimination, a result that also implies configural as well as motion feature integration.

We suggest that an accurate and complete account of *how* auditory and visual speech is integrated or interacts will require abandoning the viseme as *the* unit of visual speech perception and taking into full account the information that is actually available to the visual speech perceiver. From our perspective, acceptance of the viseme as *the* unit of visual speech perception stands in the way of understanding *how* audiovisual speech is integrated as well as *what* is integrated.

### Visual Speech Synthesis

There are many applications for a visual speech synthesizer, including its potential for use in language training, human-machine interfaces, and clinical or educational training (Massaro et al., 2012; Mattheyses and Verhelst, 2014). For example, audiovisual stimuli have been shown to promote perceptual learning of phoneme contrasts in a second language (Hazan et al., 2006), and a synthesizer could be used to provide unlimited visible speech materials for a training approach (Massaro, 2006). Our intent here was to use synthesis to probe the perceptual basis for visual phoneme identification and discrimination, and extend the work of Jiang et al. (2007) by using their model of perceptual dissimilarity.

A practical question is how much information should be engineered into synthesized visible speech? The discrimination results here suggest that phoneme synthesis based on a small set of visemes will not be adequate, because perceivers are sensitive to sub-visemic features. A recent report on concatenative visual speech synthesis also supports the importance of co-articulatory visual phonetic detail. Mattheyses et al. (2013) showed that fixed viseme units are inferior to phonemic-context-sensitive visemes. Another synthesis study found that 150 dynamic “viseme” sequences resulted in higher quality judgments than sequences based on static viseme poses (Taylor et al., 2012).

However, the synthetic visible speech tokens in the present study were not developed for a general speech synthesis application. They were generated exclusively on the basis of three-dimensional optical data that were recorded at the time the video frames were recorded and were appropriate for investigating relationships between modeled dissimilarities and discrimination based on synthesis using the same data. The more accurate identification of natural /hɑ, dƷɑ, lɑ, nɑ, rɑ/ than synthesized visible speech and the lower entropy for natural /tʃɑ, dɑ, fɑ, hɑ, lɑ, nɑ, tɑ/ can be attributed to readily identifiable weakness in this approach to synthesis.

The three-dimensional optical signals were constrained by the recording technology to be obtained from the surface of the face (Jiang et al., 2007). The retro-reflectors that were used to record the infrared flashes were relatively sparsely spaced over the face. This is a particular concern for modeling the lips. **Figure 1A** shows that the lip retro-reflectors were place around the vermillion border of the lip. However, during speech production the outer lip border does not maintain a constant relationship with the inner edge of the lips.

There were no optical markers on the tongue. Lipreaders of natural visible speech stimuli have visual access to the tongue when the mouth is open. Tongue motion is incompletely correlated with movement of the face (Yehia et al., 1998; Jiang et al., 2002), so being able to see into the mouth can afford additional speech information. The lack of the gesture that involves placement of the teeth against the lower lip likely accounts for the /fɑ/ natural stimuli being more accurately identified than the synthetic ones.

On the other hand, despite the relatively few optical channels that drove the synthesizer, identification and discrimination were quite good. The synthesizer was successful in eliminating stimulus information that was not incorporated in the perceptual model, and so its deficits were the same as the potential deficits in the perceptual model. We believe that the approach presented here, which uses the same three-dimensional optical data for synthesis and for modeling perception, can be a very useful technique for gaining a better understanding of what information is needed to generate high quality synthetic visual speech.

## Summary and Conclusion

A longstanding notion in the literature is that the perceptual categories of visual speech comprise groups of phonemes (referred to as *visemes*), such as /p, b, m/ and /f, v/, that do not have within-viseme internal perceptual structure or information. We evaluated this notion using a psychophysical discrimination paradigm that measured *d’* and response latency and found instead that discrimination was reliable within pairs of stimuli that are typically considered to be within visemes. Natural within-viseme stimulus pairs, except for /k/-/h/, were discriminable. Two of the *near* synthetic pairs were discriminable suggesting that more detailed or complete information is needed for synthesis. Nevertheless, even the results with synthetic speech point to the sensitivity of visual perceivers to even highly reduced speech information. Some evidence was obtained for both configural and dynamic visual speech features in that stimulus inversion reduced discrimination, although it remained reliable. The /v/ phoneme with its distinct articulation of the teeth against the lower lip appears to be particularly sensitive to inversion. The results reported here have direct implications for future research, theory, and practical applications. Our demonstration of sub-visemic discrimination implies that more phonetic information is available to comprehend speech, and as with auditory speech perception, visual speech perception is expected with even reduced phonetic cues. Thus, the theoretical status of visual speech is more parallel to that of auditory speech. Acceptance that there is a range of visual phoneme discriminability could lead to experiments with explicit control of visual phoneme discriminability, affording new opportunities to understand how information combines across auditory and visual speech input. Sub-visemic discrimination also implies that detailed visual phonetic information should improve results involving practical applications such as synthesized visual speech. Overall, abandoning the notion that visual speech perception relies on a small set of viseme categories seems a sound recommendation given our data.

## Conflict of Interest Statement

The authors declare that the research was conducted in the absence of any commercial or financial relationships that could be construed as a potential conflict of interest.

## Footnotes

1 Woodward and Barber (1960) carried out their discrimination experiment with consonant-vowel (CV) stimuli. Based on an *a priori* articulatory analysis, they ranked stimulus visibility from labial, to alveolar or palatal, to velar or glottal. They constructed a discrimination test that used the 22 initial English consonants and /hw/ and /aaaa/ and also presented CV versus /aaaa/ vowel contrasts. They only tested a sample of possible contrasts, which were grouped into ones thought to be “visually contrastive,” “visually similar,” or “visually equivalent.” Discrimination rankings were calculated based on subtracting the percentage of perceivers (*N* = 185) with “different” from “alike” scores for each stimulus pair, not taking into account same trials in their analyses of the results. They then designated stimulus pairs to be contrastive, similar, and equivalent along a roughly linear scale. They argued that the small set of contrasts could not explain proficient deaf lipreading and suggest that it must rely on higher-level linguistic knowledge, because the phonetic stimuli were too impoverished.

2 We cite these references merely as examples of usage that is extremely widespread. Any number of alternate citations would be appropriate for the same purpose.

## References

[B1] Arnal L. H. Morillon B. Kell C. A. Giraud A. L. (2009). Dual neural routing of visual facilitation in speech processing. *J. Neurosci.* 29 13445–13453. 10.1523/JNEUROSCI.3194-09.2009 19864557 PMC6665008

[B2] Auer E. T. Jr. (2002). The influence of the lexicon on speech read word recognition: contrasting segmental and lexical distinctiveness. *Psychon. Bull. Rev.* 9 341–347. 10.3758/BF03196291 12120798

[B3] Auer E. T. Jr. Bernstein L. E. (1997). Speechreading and the structure of the lexicon: computationally modeling the effects of reduced phonetic distinctiveness on lexical uniqueness. *J. Acoust. Soc. Am.* 102 3704–3710. 10.1121/1.420402 9407662

[B4] Auer E. T. Jr. Bernstein L. E. (2007). Enhanced visual speech perception in individuals with early-onset hearing impairment. *J. Speech Lang. Hear. Res.* 50 1157–1165. 10.1044/1092-4388(2007/080) 17905902

[B5] Bernstein L. E. (2012). “Visual speech perception,” in *AudioVisual Speech Processing* eds Vatikiotis-Bateson E. Bailly G. Perrier P. (Cambridge: Cambridge University) 21–39. 10.1017/CBO9780511843891.004

[B6] Bernstein L. E. Auer E. T. Jr. Takayanagi S. (2004). Auditory speech detection in noise enhanced by lipreading. *Speech Commun.* 44 5–18. 10.1016/j.specom.2004.10.011

[B7] Bernstein L. E. Auer E. T. Jr. Tucker P. E. (2001). Enhanced speechreading in deaf adults: can short-term training/practice close the gap for hearing adults? *J. Speech Lang. Hear. Res.* 44 5–18. 10.1044/1092-4388(2001/001) 11218108

[B8] Bernstein L. E. Demorest M. E. Tucker P. E. (2000). Speech perception without hearing. *Percept. Psychophys.* 62 233–252. 10.3758/BF03205546 10723205

[B9] Bernstein L. E. Jiang J. Pantazis D. Lu Z. L. Joshi A. (2011). Visual phonetic processing localized using speech and nonspeech face gestures in video and point-light displays. *Hum. Brain Mapp.* 32 1660–1676. 10.1002/hbm.21139 20853377 PMC3120928

[B10] Bernstein L. E. Liebenthal E. (2014). Neural pathways for visual speech perception. *Front. Neurosci.* 8:386 10.3389/fnins.2014.00386 PMC4248808 25520611

[B11] Campbell R. (2008). The processing of audio-visual speech: empirical and neural bases. *Philos. Trans. R. Soc. Lond. B Biol. Sci.* 363 1001–1010. 10.1098/rstb.2007.2155 17827105 PMC2606792

[B12] Campbell R. (2011). Speechreading and the Bruce-Young model of face recognition: early findings and recent developments. *Br. J. Psychol.* 102 704–710. 10.1111/j.2044-8295.2011.02021.x 21988379

[B13] Catford J. C. (1977). *Fundamental Problems in Phonetics*. Bloomington, IN: Indiana University.

[B14] Conklin E. S. (1917). A method for the determination of relative skill in lip-reading. *Volta Rev.* 19 216–219.

[B15] Czigler I. (2007). Visual mismatch negativity: violation of nonattended environmental regularities. *J. Psychophysiol.* 21 224–230. 10.1027/0269-8803.21.34.224

[B16] Demorest M. E. Bernstein L. E. (1992). Sources of variability in speechreading sentences: a generalizability analysis. *J. Speech Hear. Res.* 35 876–891. 10.1044/jshr.3504.876 1405543

[B17] Demorest M. E. Bernstein L. E. DeHaven G. P. (1996). Generalizability of speechreading performance on nonsense syllables, words, and sentences: subjects with normal hearing. *J. Speech Hear. Res.* 39 697–713. 10.1044/jshr.3904.697 8844551

[B18] Desai R. Liebenthal E. Waldron E. Binder J. R. (2008). Left posterior temporal regions are sensitive to auditory categorization. *J. Cogn. Neurosci.* 20 1174–1188. 10.1162/jocn.2008.20081 18284339 PMC3350814

[B19] Eberhardt S. P. Bernstein L. E. Demorest M. E. Goldstein M. H. Jr. (1990). Speechreading sentences with single-channel vibrotactile presentation of voice fundamental frequency. *J. Acoust. Soc. Am.* 88 1274–1285. 10.1121/1.399704 2146296

[B20] Feld J. Sommers M. (2011). There goes the neighborhood: lipreading and the structure of the mental lexicon. *Speech Commun.* 53 220–228. 10.1016/j.specom.2010.09.003 21170172 PMC3002260

[B21] Files B. T. Auer E. T. Jr. Bernstein L. E. (2013). The visual mismatch negativity elicited with visual speech stimuli. *Front. Hum. Neurosci.* 7:371 10.3389/fnhum.2013.00371 PMC3712324 23882205

[B22] Fisher C. G. (1968). Confusions among visually perceived consonants. *J. Speech Hear. Res.* 11 796–804. 10.1044/jshr.1104.796 5719234

[B23] Fisher C. G. (1969). The visibility of terminal pitch contour. *J. Speech Hear. Res.* 12 379–382. 10.1044/jshr.1202.379 5808865

[B24] Fox C. J. Iaria G. Barton J. J. (2009). Defining the face processing network: optimization of the functional localizer in fMRI. *Hum. Brain Mapp.* 30 1637–1651. 10.1002/hbm.20630 18661501 PMC6870735

[B25] Gold J. M. Mundy P. J. Tjan B. S. (2012). The perception of a face is no more than the sum of its parts. *Psychol. Sci.* 23 427–434. 10.1177/0956797611427407 22395131 PMC3410436

[B26] Grant K. W. Walden B. E. Seitz P. F. (1998). Auditory-visual speech recognition by hearing-impaired subjects: consonant recognition, sentence recognition, and auditory-visual integration. *J. Acoust. Soc. Am.* 103(5 Pt 1) 2677–2690. 10.1121/1.422788 9604361

[B27] Grant K. W. Walden B. E. Summers V. Leek M. R. (2013). Introduction: auditory models of suprathreshold distortion in persons with impaired hearing. *J. Am. Acad. Audiol.* 24 254–257. 10.3766/jaaa.24.4.2 23636207

[B28] Hazan V. Sennema A. Faulkner A. Ortega-Llebaria M. (2006). The use of visual cues in the perception of non-native consonant contrasts. *J. Acoust. Soc. Am.* 119 1740–1751. 10.1121/1.2166611 16583916

[B29] Hickok G. Poeppel D. (2007). The cortical organization of speech processing. *Nat. Rev. Neurosci.* 8 393–402. 10.1038/nrn2113 17431404

[B30] Hnath-Chisolm T. Kishon-Rabin L. (1988). Tactile presentation of voice fundamental frequency as an aid to the perception of speech pattern contrasts. *Ear Hear.* 9 329–334. 10.1097/00003446-198812000-00009 3220185

[B31] Iverson P. Bernstein L. E. Auer E. T. Jr. (1998). Modeling the interaction of phonemic intelligibility and lexical structure in audiovisual word recognition. *Speech Commun.* 26 45–63. 10.1016/S0167-6393(98)00049-1

[B32] Jerger S. Damian M. F. Spence M. J. Tye-Murray N. Abdi H. (2009). Developmental shifts in children’s sensitivity to visual speech: a new multimodal picture-word task. *J. Exp. Child Psychol.* 102 40–59. 10.1016/j.jecp.2008.08.002 18829049 PMC2612128

[B33] Jesse A. McQueen J. M. (2014). Suprasegmental lexical stress cues in visual speech can guide spoken-word recognition. *Q. J. Exp. Psychol.* 67 793–808. 10.1080/17470218.2013.834371 24134065

[B34] Jiang J. Alwan A. Keating P. Auer E. T. Jr. Bernstein L. E. (2002). On the relationship between face movements, tongue movements, and speech acoustics. *EURASIP J. Appl. Signal Process.* 2002 1174–1188. 10.1155/S1110865702206046

[B35] Jiang J. Aronoff J. M. Bernstein L. E. (2008). Development of a visual speech synthesizer via second-order isomorphism. *Paper Presented at the International Conference on Acoustics, Speech and Signal Processing, 2008. ICASSP 2008*. Las Vegas, NV: IEEE 10.1109/icassp.2008.4518700

[B36] Jiang J. Auer E. T. Jr. Alwan A. Keating P. A. Bernstein L. E. (2007). Similarity structure in visual speech perception and optical phonetic signals. *Percept. Psychophys.* 69 1070–1083. 10.3758/BF03193945 18038946

[B37] Jiang J. Bernstein L. E. (2011). Psychophysics of the McGurk and other audiovisual speech integration effects. *J. Exp. Psychol. Hum. Perform. Percept.* 37 1193–1209. 10.1037/a0023100 PMC3149717 21574741

[B38] Jiang X. Rosen E. Zeffiro T. VanMeter J. Blanz V. Riesenhuber M. (2006). Evaluation of a shape-based model of human face discrimination using fMRI and behavioral techniques. *Neuron* 50 159–172. 10.1016/j.neuron.2006.03.012 16600863

[B39] Kailath T. Sayed A. H. Hassibi B. (2000). *Linear Estimation*. Upper Saddle River, NJ: Prentice Hall.

[B40] Kimura M. Schroger E. Czigler I. (2011). Visual mismatch negativity and its importance in visual cognitive sciences. *Neuroreport* 22 669–673. 10.1097/WNR.0b013e32834973ba 21878790

[B41] Kruskal J. B. Wish M. (1978). *Multidimensional Scaling*. Berverly Hills, CA: Sage.

[B42] Kyle F. E. Campbell R. Mohammed T. Coleman M. Macsweeney M. (2013). Speechreading development in deaf and hearing children: introducing the test of child speechreading. *J. Speech Lang. Hear. Res.* 56 416–426. 10.1044/1092-4388(2012/12-0039) 23275416 PMC4920223

[B43] Lalonde K. Holt R. F. (2014). Preschoolers benefit from visually-salient speech cues. *J. Speech Lang. Hear. Res.* 58 135–150. 10.1044/2014_jslhr-h-13-0343 25322336 PMC4712850

[B44] Lansing C. R. McConkie G. W. (1999). Attention to facial regions in segmental and prosodic visual speech perception tasks. *J. Speech Lang. Hear. Res.* 42 526–539. 10.1044/jslhr.4203.526 10391620

[B45] Liberman A. M. Harris K. S. Hoffman H. S. Griffith B. C. (1957). The discrimination of speech sounds within and across phoneme boundaries. *J. Exp. Psychol. Hum. Percept. Perform.* 54 358–368. 10.1037/h0044417 13481283

[B46] Lucero J. C. Munhall K. G. (2008). Analysis of facial motion patterns during speech using a matrix factorization algorithm. *J. Acoust. Soc. Am.* 124 2283–2290. 10.1121/1.2973196 19062866 PMC2736716

[B47] Lyxell B. Ronnberg J. Andersson J. Linderoth E. (1993). Vibrotactile support: initial effects on visual speech perception. *Scand. Audiol.* 22 179–183. 10.3109/01050399309047465 8210957

[B48] Ma J. Cole R. Pellom B. Ward W. Wise B. (2006). Accurate visible speech synthesis based on concatenating variable length motion capture data. *IEEE Trans. Vis. Comput. Graph.* 12 266–276. 10.1109/TVCG.2006.18 16509385

[B49] MacEachern E. (2000). On the visual distinctiveness of words in the English lexicon. *J. Phon.* 28 367–376. 10.1006/jpho.2000.0119

[B50] MacLeod A. Summerfield Q. (1987). Quantifying the contribution of vision to speech perception in noise. *Br. J. Audiol.* 21 131–141. 10.3109/03005368709077786 3594015

[B51] Macmillan N. A. Creelman C. D. (1991). *Detection Theory : A User’s Guide*. New York, NY: Cambridge University Press.

[B52] Massaro D. W. (2006). “A computer-animated tutor for language learning: research and applications,” in *Advances in the Spoken Language Development of Deaf and Hard-of-Hearing Children* eds Spencer P. E. Marshark M. (New York, NY: Oxford University Press) 212–243.

[B53] Massaro D. W. Cohen M. M. (1983). *Speech Perception by Eear and Eye: A Paradigm for Psychological Inquiry*. London: Erlbaum.

[B54] Massaro D. W. Cohen M. M. (1996). Perceiving speech from inverted faces. *Percept. Psychophys.* 58 1047–1065. 10.3758/BF03206832 8920841

[B55] Massaro D. W. Cohen M. M. Gesi A. T. (1993). Long-term training, transfer, and retention in learning to lipread. *Percept. Psychophys.* 53 549–562. 10.3758/BF03205203 8332424

[B56] Massaro D. W. Cohen M. M. Tabain M. Beskow J. (2012). “Animated speech: research progress and applications,” in *Audiovisual Speech Processing* eds Clark R. B. Perrier J. P. Vatikiotis-Bateson E. (Cambridge: Cambridge University) 246–272. 10.1017/cbo9780511843891.014

[B57] Matchin W. Groulx K. Hickok G. (2013). Audiovisual speech integration does not rely on the motor system: evidence from articulatory suppression, the McGurk effect, and fMRI. *J. Cogn. Neurosci.* 26 606–620. 10.1162/jocn_a_00515 24236768 PMC5241269

[B58] Mattheyses W. Latacz L. Verhelst W. (2013). Comprehensive many-to-many phoneme-to-viseme mapping and its application for concatenative visual speech synthesis. *Speech Commun.* 55 857–876. 10.1016/j.specom.2013.02.005

[B59] Mattheyses W. Verhelst W. (2014). Audiovisual speech synthesis: an overview of the state-of-the-art. *Speech Commun.* 66 182–217. 10.1016/j.specom.2014.11.001

[B60] Mattys S. L. Bernstein L. E. Auer E. T. Jr. (2002). Stimulus-based lexical distinctiveness as a general word-recognition mechanism. *Percept. Psychophys.* 64 667–679. 10.3758/BF03194734 12132766

[B61] Maurer D. Grand R. L. Mondloch C. J. (2002). The many faces of configural processing. *Trends Cogn. Sci.* 6 255–260. 10.1016/S1364-6613(02)01903-4 12039607

[B62] Morey R. D. (2008). Confidence intervals from normalized data: a correction to cousineau (2005). *Tutor. Quant. Methods Psychol.* 4 61–64.

[B63] Munhall K. G. Jones J. A. Callan D. E. Kuratate T. Vatikiotis-Bateson E. (2004). Visual prosody and speech intelligibility: head movement improves auditory speech perception. *Psychol. Sci.* 15 133–137. 10.1111/j.0963-7214.2004.01502010.x 14738521

[B64] Owens E. Blazek B. (1985). Visemes observed by hearing-impaired and normal hearing adult viewers. *J. Speech Hear. Res.* 28 381–393. 10.1044/jshr.2803.381 4046579

[B65] Pazo-Alvarez P. Cadaveira F. Amenedo E. (2003). MMN in the visual modality: a review. *Biol. Psychol.* 63 199–236. 10.1016/S0301-0511(03)00049-8 12853168

[B66] Pitcher D. Dilks D. D. Saxe R. R. Triantafyllou C. Kanwisher N. (2011). Differential selectivity for dynamic versus static information in face-selective cortical regions. *Neuroimage* 56 2356–2363. 10.1016/j.neuroimage.2011.03.067 21473921

[B67] Raphael L. J. (1971). Preceding vowel duration as a cue to the perception of the voicing characteristic of word-final consonants in American English. *J. Acoust. Soc. Am.* 51 1296–1303. 10.1121/1.1912974 5032946

[B68] Rauschecker J. P. Scott S. K. (2009). Maps and streams in the auditory cortex: nonhuman primates illuminate human speech processing. *Nat. Neurosci.* 12 718–724. 10.1038/nn.2331 19471271 PMC2846110

[B69] Richler J. J. Mack M. L. Palmeri T. J. Gauthier I. (2011). Inverted faces are (eventually) processed holistically. *Vision Res.* 51 333–342. 10.1016/j.visres.2010.11.014 21130798

[B70] Rosenblum L. D. Johnson J. A. Saldana H. M. (1996). Point-light facial displays enhance comprehension of speech in noise. *J. Speech Hear. Res.* 39 1159–1170. 10.1044/jshr.3906.1159 8959601

[B71] Rosenblum L. D. Saldana H. M. (1996). An audiovisual test of kinematic primitives for visual speech perception. *J. Exp. Psychol. Hum. Percept. Perform.* 22 318–331. 10.1037/0096-1523.22.2.318 8934846

[B72] Rosenblum L. D. Yakel D. A. Green K. P. (2000). Face and mouth inversion effects on visual and audiovisual speech perception. *J. Exp. Psychol. Hum. Percept. Perform.* 26 806–819. 10.1037/0096-1523.26.2.806 10811177

[B73] Ross L. A. Saint-Amour D. Leavitt V. M. Javitt D. C. Foxe J. J. (2007). Do you see what I am saying? Exploring visual enhancement of speech comprehension in noisy environments. *Cereb. Cortex* 17 1147–1153. 10.1093/cercor/bhl024 16785256

[B74] Scarborough R. Keating P. Baroni M. Cho T. Mattys S. Alwan A. (2007). *Optical Cues to the Visual Perception of Lexical and Phrasal Stress in English. UCLA Working Papers in Phonetics* 105 118–124. Available at: http://escholarship.org/uc/item/4gk6008p

[B75] Schwartz J. L. Berthommier F. Savariaux C. (2004). Seeing to hear better: evidence for early audio-visual interactions in speech identification. *Cognition* 93 B69–B78. 10.1016/j.cognition.2004.01.006 15147940

[B76] Shannon C. E. (1948). A mathematical theory of communication. *Bell Syst. Technical J.* 27 379–423. 10.1002/j.1538-7305.1948.tb01338.x

[B77] Stein B. E. Meredith M. A. (1990). Multisensory integration. *Ann. N. Y. Acad. Sci.* 608 51–70. 10.1111/j.1749-6632.1990.tb48891.x 2075959

[B78] Sumby W. H. Pollack I. (1954). Visual contribution to speech intelligibility in noise. *J. Acoust. Soc. Am.* 26 212–215. 10.1121/1.1907309

[B79] Susilo T. McKone E. Edwards M. (2010). Solving the upside-down puzzle: why do upright and inverted face aftereffects look alike? *J. Vis.* 10 1–16. 10.1167/10.13.1 21149314

[B80] Taylor S. L. Mahler M. Theobald B.-J. Matthews I. (2012). Dynamic units of visual speech. *Paper Presented at the Proceedings of the 11th ACM SIGGRAPH / Eurographics conference on Computer Animation* Lausanne 10.2312/SCA/SCA12/275-284

[B81] Thomas S. M. Jordan T. R. (2004). Contributions of oral and extraoral facial movement to visual and audiovisual speech perception. *J. Exp. Psychol. Hum. Percept. Perform.* 30 873–888. 10.1037/0096-1523.30.5.873 15462626

[B82] Tjan B. S. Chao E. Bernstein L. E. (2014). A visual or tactile signal makes auditory speech detection more efficient by reducing uncertainty. *Eur. J. Neurosci.* 39 1323–1331. 10.1111/ejn.12471 24400652 PMC3997613

[B83] Tye-Murray N. Hale S. Spehar B. Myerson J. Sommers M. S. (2014). Lipreading in school-age children: the roles of age, hearing status, and cognitive ability. *J. Speech Lang. Hear. Res.* 57 556–565. 10.1044/2013_jslhr-h-12-0273 24129010 PMC5736322

[B84] Utley J. (1946). A test of lip reading ability. *J. Speech Hear. Disord.* 11 109–116. 10.1044/jshd.1102.109 20986556

[B85] Valentine T. (1988). Upside-down faces: a review of the effect of inversion upon face recognition. *Br. J. Psychol.* 79(Pt 4) 471–491. 10.1111/j.2044-8295.1988.tb02747.x 3061544

[B86] Walden B. E. Montgomery A. A. Prosek R. A. (1987). Perception of synthetic visual consonant-vowel articulations. *J. Speech Hear. Res.* 30 418–424. 10.1044/jshr.3003.418 3669649

[B87] Walden B. E. Prosek R. A. Montgomery A. A. Scherr C. K. Jones C. J. (1977). Effects of training on the visual recognition of consonants. *J. Speech Hear. Res.* 20 130–145. 10.1044/jshr.2001.130 846196

[B88] Walden B. E. Prosek R. A. Worthington D. W. (1975). Auditory and audiovisual feature transmission in hearing-impaired adults. *J. Speech Hear. Res.* 18 272–280. 10.1044/jshr.1802.272 4836044

[B89] Winkler I. Czigler I. (2012). Evidence from auditory and visual event-related potential (ERP) studies of deviance detection (MMN and vMMN) linking predictive coding theories and perceptual object representations. *Int. J. Psychophysiol.* 83 132–143. 10.1016/j.ijpsycho.2011.10.001 22047947

[B90] Woodward M. F. Barber C. G. (1960). Phoneme perception in lipreading. *J. Speech Hear. Res.* 3 212–222. 10.1044/jshr.0303.212 13845910

[B91] Xue J. Borgstrom J. Jiang J. Bernstein L. E. Alwan A. (2006). Acoustically-driven talking face synthesis using dynamic Bayesian networks. *Paper Presented at the Proceedings of IEEE International Conference on Multimedia and Expo (ICME)* Toronto 10.1109/icme.2006.262743

[B92] Yehia H. Rubin P. Vatikiotis-Bateson E. (1998). Quantitative association of vocal-tract and facial behavior. *Speech Commun.* 26 23–43. 10.1016/s0167-6393(98)00048-x

[B93] Yovel G. Kanwisher N. (2005). The neural basis of the behavioral face-inversion effect. *Curr. Biol.* 15 2256–2262. 10.1016/j.cub.2005.10.072 16360687

